# Integrated Analysis of MATH-Based Subtypes Reveals a Novel Screening Strategy for Early-Stage Lung Adenocarcinoma

**DOI:** 10.3389/fcell.2022.769711

**Published:** 2022-02-08

**Authors:** Chang Li, Chen Tian, Yulan Zeng, Jinyan Liang, Qifan Yang, Feifei Gu, Yue Hu, Li Liu

**Affiliations:** ^1^ Cancer Center, Union Hospital, Tongji Medical College, Huazhong University of Science and Technology, Wuhan, China; ^2^ Department of Ultrasound, Union Hospital, Tongji Medical College, Huazhong University of Science and Technology, Wuhan, China

**Keywords:** lung adenocarcinoma, diagnosis, mutant allele tumor heterogeneity, machine learning, classification, immunotherapy, drug sensitivity

## Abstract

Lung adenocarcinoma (LUAD) is a frequently diagnosed cancer type, and many patients have already reached an advanced stage when diagnosed. Thus, it is crucial to develop a novel and efficient approach to diagnose and classify lung adenocarcinoma at an early stage. In our study, we combined *in silico* analysis and machine learning to develop a new five-gene–based diagnosis strategy, which was further verified in independent cohorts and *in vitro* experiments. Considering the heterogeneity in cancer, we used the MATH (mutant-allele tumor heterogeneity) algorithm to divide patients with early-stage LUAD into two groups (C1 and C2). Specifically, patients in C2 had lower intratumor heterogeneity and higher abundance of immune cells (including B cell, CD4 T cell, CD8 T cell, macrophage, dendritic cell, and neutrophil). In addition, patients in C2 had a higher likelihood of immunotherapy response and overall survival advantage than patients in C1. Combined drug sensitivity analysis (CTRP/PRISM/CMap/GDSC) revealed that BI-2536 might serve as a new therapeutic compound for patients in C1. In order to realize the application value of our study, we constructed the classifier (to classify early-stage LUAD patients into C1 or C2 groups) with multiple machine learning and bioinformatic analyses. The 21-gene–based classification model showed high accuracy and strong generalization ability, and it was verified in four independent validation cohorts. In summary, our research provided a new strategy for clinicians to make a quick preliminary assisting diagnosis of early-stage LUAD and make patient classification at the intratumor heterogeneity level. All data, codes, and study processes have been deposited to Github and are available online.

## Introduction

Non–small cell lung cancer (NSCLC) is the most common variety of lung cancer, which is the leading cause of cancer-related death worldwide (Bray et al., 2018; Duma et al., 2019). Lung adenocarcinoma (LUAD) is the major histological type of NSCLC. According to previous studies, lung adenocarcinoma is often heterogenous. Despite that great advance in the treatment of LUAD has been made in the past few decades, the 5-years survival is still not satisfactory (Baba et al., 2012; Zappa and Mousa, 2016; Shroff et al., 2018). Due to the mild early symptoms, most patients have already reached an advanced stage when diagnosed, and it results in poor long-term overall survival. Thus, it is urgent to develop efficient biomarkers or signatures that could be used in the diagnosis of LUAD. Meanwhile, understanding the heterogeneity in early-stage lung adenocarcinoma is critical to select and develop more effective treatment.

Intratumor heterogeneity refers to the subclones of diverse genetic background within a tumor, and it is increasingly identified as a key factor in the treatment failure of human cancers. With the rise of next-generation sequencing and machine learning applications in oncology (Cho et al., 2020; Li et al., 2021; Wang et al., 2021), computational approaches (such as ABSOLUTE) were developed to quantify intratumor heterogeneity based on biological information (Thorsson et al., 2018). MATH (mutant-allele tumor heterogeneity) is a quantitative approach to depict ITH based on variant allele frequency information. In brief, mutant-allele fractions among genomic locus-bearing somatic mutations will be widely distributed in the tumors with distinct subclones, and MATH is a quantitative assessment to normalize the width of such distribution (Rocco, 2015; Ran et al., 2020). In this study, we used MATH to quantify ITH of early-stage LUAD patients and intended to find MATH-based subtypes. We have explored not only the characteristics of these novel subtypes of LUAD but also the potential treatment for LUAD patients at an early stage.

## Materials and Methods

### Data Pre-processing

RNA sequencing of combined TCGA and GTEx data (free of computational batch effects) and TCGA-LUAD (lung adenocarcinoma) data were downloaded from UCSC Xena (https://xenabrowser.net/datapages/). Relevant clinical information was also collected from the UCSC Xena browser. The expression data derived from TCGA database were pre-processed by the following steps: 1) removing samples without clinical information; 2) preserving early-stage (stage I and stage II) samples; and 3) expression data were TPM-normalized and genes with log2 (TPM+1) >0 were preserved. Additionally, copy number alteration and somatic mutation MAF data were downloaded from TCGA data portal (https://portal.gdc.cancer.gov/).

The independent validation cohorts (including GSE30219, GSE31210, GSE50081, and GSE72094) were downloaded from GEO (Gene Expression Omnibus, http://www.ncbi.nlm.nih.gov/geo/). Detailed information of the study cohorts is shown in the *Supplementary Material*.

### Construction of the Diagnosis Model

Differential gene expression analysis between non-cancerous lung tissues and lung adenocarcinoma tissues was conducted in TCGA-GTEx, GSE30219, and GSE31210 cohorts. DEGs were identified by having log2fc > 1 and fdr <0.05. Robust rank aggregation (RRA) was used to identify overlapping DEGs (Gan et al., 2020), which were used to develop the diagnosis model. The patients in TCGA-GTEx cohorts were randomly assigned to the training and validation group at a ratio of 7:3. The other datasets (GSE30219 and GSE31210) were used as two independent validation cohorts. Machine learning including elastic net regression (ElasticNet, binomial, alpha = 0.9), Random Forest and Bortua (RFB, default), Support Vector Machine-Recursive Feature Elimination (SVM-RFE, svmRFE function in R, k = 5, halve. above = 100), and eXtreme Gradient Boosting (XGBoost, xgboost function in R, default) was performed to identify the most important predictors. The expression of the overlapping DEGs was used as the input variable, and the status of the tissues (tumor or non-cancerous lung tissue, 1 or 0) was set as the response variable. In the training group, the intersect genes identified by ElasticNet, RFB, SVM-RFE, and XGBoost were collected, and logistic regression analysis was performed on these genes to develop the diagnosis model. The performance of the diagnosis model (in the training and validation group, as well as two independent validation cohorts) was evaluated by receiver operating characteristic (ROC) curves and AUC values.

### Real-Time Quantitative Polymerase Chain Reaction

Total RNA was extracted with TRIzol reagent (Takara), and the synthesis of cDNA was conducted with the qPCR RT Master Mix (Toyobo). To detect the expression of target genes, PCR was performed with the SYBR Green Real-Time PCR kit (Takara) on the StepOnePlus™ Real-Time PCR System (ABI) based on the manufacturer’s instructions. GAPDH was selected as the internal control, and the relative expression levels were determined by comparative Ct (target gene Ct minus GAPDH Ct). Sequences of the primers are listed as follows:

B3GNT3-F: CTT​GCT​GTC​CCG​CTT​CAC.

B3GNT3-R: GAG​GCA​GGC​TTC​AGT​CCC.

GALNT7-F: GAA​TCG​CAG​GCA​TTA​CCA.

GALNT7-R: AAG​CCT​CTG​ATT​TCT​CCC.

PLEK2-F: CAC​GGT​GGT​GAA​ACA​AGG.

PLEK2-R: CAG​TGG​GAA​CGC​CAT​TAT.

GAPDH-F: GAG​TCA​ACG​GAT​TTG​GTC​GT.

GAPDH-R: GAC​AAG​CTT​CCC​GTT​CTC​AG.

### CCK-8 Assay

The cells were seeded in 96-well plates at 2000 cells per well (A549) or 1800 cells per well (H1299) with complete medium. After 1, 2, 3, and 4 days of culture, the CCK-8 kit (Dojindo) was used to detect cell proliferation, and the absorbance was read at 450 nm.

### Calculation of the MATH Value and Gene Expression–Based Stemness Index

Mutant-allele tumor heterogeneity (MATH) is a quantitative strategy to quantify the dispersion of allele frequencies of somatic mutations based on whole-exome sequencing data (Rocco, 2015; McDonald et al., 2019). MATH score was calculated by “inferHeterogenetiy” function (“maftools” package in R). In this study, MATH score was used to measure intratumor heterogeneity (ITH), and we compared the MATH score with the ABSOLUTE score (obtained from Thorsson V et al. study) to ensure it could reflect the ITH accurately.

To calculate the mRNAsi, Malta et al. built a predictive model using one-class logistic regression (OCLR) on the Progenitor Cell Biology Consortium cohort to calculate stemness signatures, which contains the gene expression profile of 11,774 genes (Lian et al., 2019). We applied the stemness signature to calculate the mRNAsi index for patients in our study using Spearman’s correlation analysis.

### Identification of the MATH-Based Molecular Subtypes of Lung Adenocarcinoma at an Early Stage

To classify patients into the MATH-based subtypes, survival analysis was performed, and X-tile was used to determine the optimal cutoff of the MATH score (Li et al., 2016). Afterward, the high-MATH group and low-MATH group were generated, and differential gene expression analysis was performed to find DEGs differentially between the two groups. Unsupervised consensus clustering (kmeans, “ConsensusClusterPlus” package in R) (Wang et al., 2021) based on these DEGs was conducted to explore a novel classification of lung adenocarcinoma: the MATH-based subtypes. This procedure was repeated 1,000 times and sampled 80% in each iteration to ensure classification stability.

### Calculation of CNA Burden, TMB, and Immunological Characteristics

CNA (copy number alteration) data of TCGA cohort was obtained from TCGA data portal. Amplified or deleted genomes in the whole genome were identified by GISTIC 2.0. The burden of copy number loss or gain was defined as the total number of genes with copy number changes in each sample at the arm and focal levels (Shen R et al., 2019).

TMB was defined as the number of non-synonymous alterations per MB of the genome. Non-synonymous mutations were defined as “Frame_Shift_Del”, “Frame_Shift_Ins”, “Missense_Mutation”, “Nonsense_Mutation”, “Splice_Site”, “In_Frame_Del”, “In_Frame_Ins”, “Translation_Start_Site”, and “Nonstop_Mutation”. The exome size was defined as 38 Mb as described in the previous study. TMB was calculated by this formula: TMB = non-synonymous mutations/exome size (38 Mb) (Wang et al., 2019).

The abundance of six immune cells (including B cell, macrophage, dendritic cell, neutrophil, T cell CD4, and T cell CD8) was calculated by TIMER (Li et al., 2020). The abundance of intratumoral immune and stromal cells was predicted using the ESTIMATE algorithm (“ESTIMATE” package in R) (Yoshihara et al., 2013). In addition, the enrichment level of 29 immune signatures (Yang et al., 2018), which represent the immune activity of tumors, and three signatures (Messina et al., 2012; Ayers et al., 2017; Jiang et al., 2018), which represent the immunotherapy response, was quantified by ssGSEA (single-sample gene set enrichment analysis).

### Prediction of TIDE Score and Immunotherapy Response

TIDE (tumor immune dysfunction and exclusion, http://tide.dfci.harvard.edu/) score, which was developed based on the mechanism of tumor immune escape, inducing T cell dysfunction in tumors with high infiltration of cytotoxic T lymphocytes (CTL) and inhibiting T cell infiltration in tumors with low CTL level, was used to predict the clinical response to immunotherapy of patients involved in our study (Jiang et al., 2018). The gene expression value had been normalized before calculation. Then, subclass mapping was processed to realize the prediction of clinical response to anti-PD1 or anti-CTLA4 therapy (Hubble et al., 2009) (“SubMap” modules in GenePattern, https://cloud.genepattern.org/gp/pages/index.jsf. A published dataset with melanoma that responded to immunotherapy was set as the reference; custom settings were set as default).

### Construction and Validation of the MATH-Based Subtype Classifier

The TCGA-LUAD patients at an early stage were randomly assigned to the training and validation group at a ratio of 7:3. GSE30219, GSE31210, GSE50081, and GSE72094 were used as external independent validation cohorts. Machine learning algorithms, including ElasticNet, RFB, SVM-RFE, and XGBoost, were performed to identify the most important predictors. The expression of the DEGs was used as the input variable, and the subtype of the sample (subtype I or subtype II, 0 or 1) was set as the response variable. In the training group, the intersect genes identified by ElasticNet, RFB, SVM-RFE, and XGBoost were collected, and logistic regression analysis was performed on these genes to develop the classification model. The model was tested in the validation group. In order to test our classifier generalization ability in the external independent validation cohorts (GSE30219, GSE31210, GSE50081, and GSE72094), we performed the following analysis: 1) performing the same k-means clustering in each cohort based on the same DEGs; 2) comparing the expression profile of the subtypes we defined in TCGA cohort with k-means clustering results in each validation cohort (by using “SubMap” module in GenePattern); and 3) determining the clustering subtypes in the validation cohorts. The performance of the classifier was investigated by AUC values.

### Drug Sensitivity Analysis

Three approaches were used to conduct drug sensitivity analysis. First, we used the CTRP (Cancer Therapeutics Response Portal) and PRISM (Profiling Relative Inhibition Simultaneously in Mixtures) to generate drug sensitivity data (Rees et al., 2016; Corsello et al., 2020). Both databases used AUC values as a measure of drug/compound sensitivity. Compounds with missing AUC values > 20% of the samples and cell lines were excluded. The “pRRophetic” package was used to predict the candidate potential drugs in each MATH-based subtype. Then, we predicted the candidate potential drug response for each sample based on the GDSC (the Genomics of Drug Sensitivity in Cancer) database (Yang et al., 2013). IC_50_ (the samples’ half-maximal inhibitory concentration) was estimated based on the GDSC dataset. In addition, we used the CMap (Connectivity Map) database to explore the drugs targeting the genes associated with the MATH-based subtypes (Musa et al., 2018). We queried the CMap database and selected the compound with a negative enrichment score and *p* < 0.05. The compound overlapping in the results of CTRP/PRISM, GDSC, and CMap analyses was considered important and may serve as a potential treatment for the certain subtype.

### Statistical Analysis

The χ^2^ test was utilized to evaluate the association between subtypes and mutations. The Shapiro–Wilk normality test was used to test the normality of data. Correlations were analyzed using Spearman’s correlation. Statistical analyses were conducted using Kruskal–Wallis, Wilcoxon, or Student’s t test. Differences were thought to be significant at *p* < 0.05. All analyses were performed in R (Version: 3.5.3). All data, codes, and workflow have been deposited to Github.

## Result

### Establishment of the Early-Stage LUAD Diagnostic Model

**FIGURE 1:**
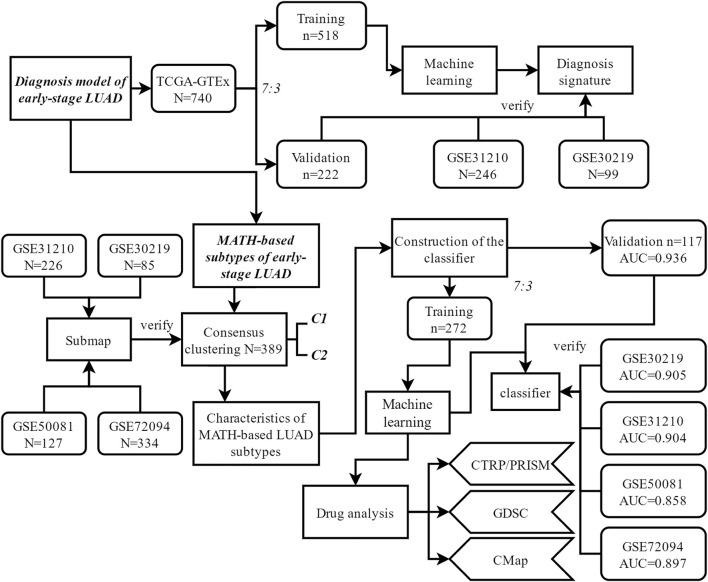
Flowchart of this study.

**FIGURE 2:**
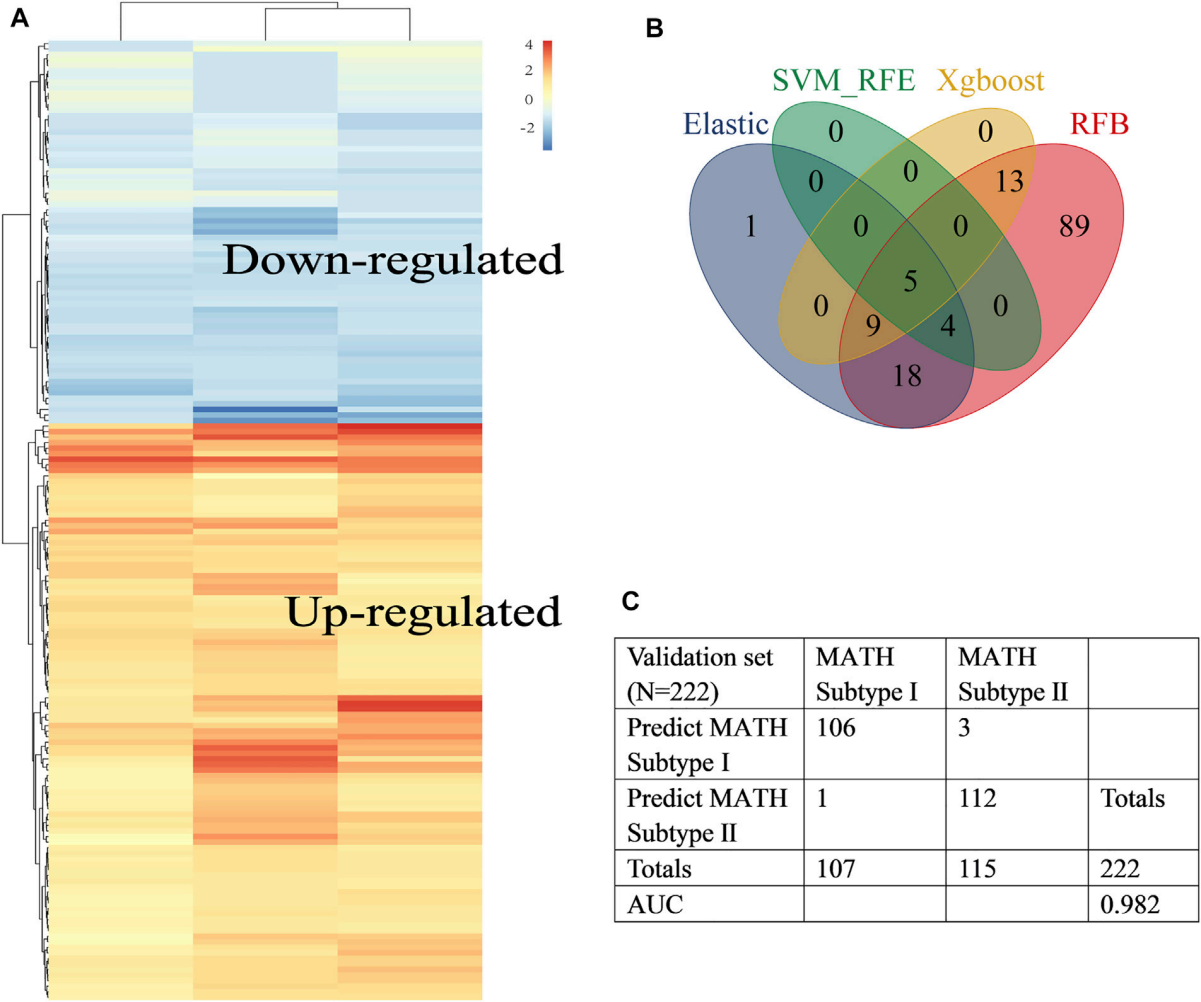
Construction of the early-stage LUAD diagnosis model. **(A)** Heat map showed differentially expressed genes in tumor tissues (abs logFC>1, fdr<0.05) identified by RRA. Rows represent DEGs and columns represent different datasets. **(B)** Venn diagram identified five diagnosis genes shared by ElasticNet, SVM-RFE, RFB, and XGBoost machine learning. **(C)**. Confusion matrix of binary result of the diagnosis model for the internal validation cohort.

The general workflow of this study is shown in Figure 1. Differential analysis between early-stage lung adenocarcinoma tissues and non-cancerous lung tissues was performed with Limma package, and a total of 173 DEGs (differentially expressed genes) were screened *via* robust rank aggregation (Figure 2A). In order to investigate the diagnostic method for early-stage LUAD, the TCGA-GTEx cohort was divided into training and validation cohorts, and two cohorts (GSE30219 and GSE31210) were used as the external validation sets (Table 1, Supplementary Table S1). As described in *Materials and Methods*, four machine learning algorithms (including ElasticNet, RFB, SVM-RFE, and XGBoost) were applied in TCGA-GTEx training set (Supplementary Figure S1A-F). Among the 173 DEGs, five genes (Supplementary Table S2, including B3GNT3, PLEK2, GALNT7, GRK5, and SLC39A8) were found overlapping in different ML methods (Figure 2B, Supplementary Table S3). The combination of the five genes was analyzed using logistic regression to generate the diagnosis model for early-stage LUAD. The confusion matrix for TCGA-GTEx validation set and two external validation sets (GSE30219 and GSE31210) were shown in Figure 2C and Supplementary Figure S1G, with AUCs of 0.982, 0.817, and 0.850, respectively.

**TABLE 1:** Clinical information of early-stage LUAD patients in TCGA cohort.

Characteristics		No of.cases (N = 389)
Gender	Female	213
	Male	176
Primary therapy outcome	Complete remission/response	236
	Partial remission/response	2
	Stable disease	19
	Progressive disease	36
	Unknown	96
Neoadjuvant treatment	No	388
	Yes	1
Stage	Stage I	271
	Stage II	118

**FIGURE 3:**
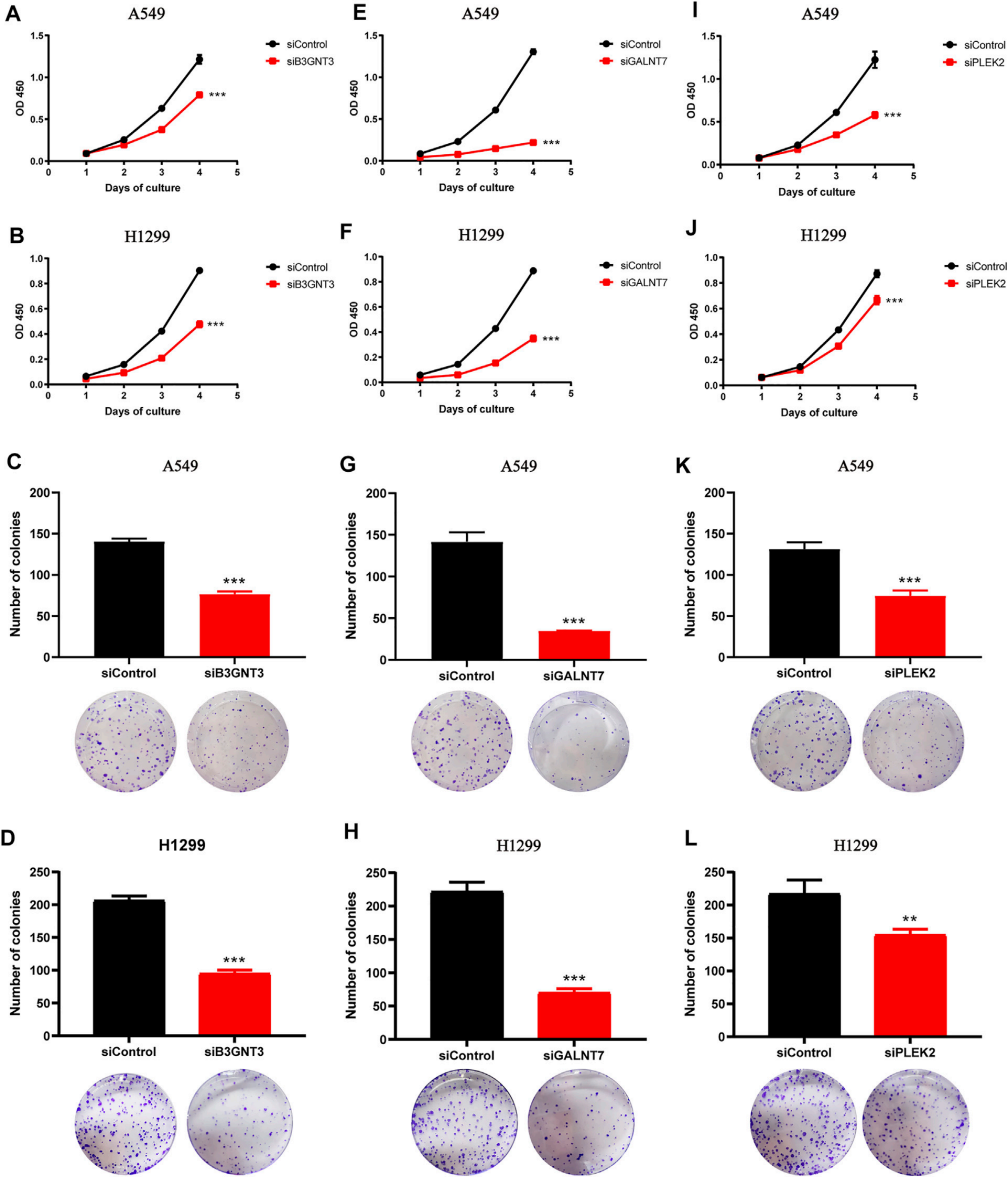
Downregulation of B3GNT3, GALNT7, or PLEK2 suppresses tumorigenesis in lung cancer. **(A–B)** CCK-8 assay revealed that B3GNT3 silencing impaired cell proliferation in A549 (A) and H1299 (B) (n = 5). **(C–D)** B3GNT3 depletion significantly weakened colony-forming capacity of A549 (C) and H1299 (D) (n = 3). **(E–H)** GALNT7. **(I–L)** PLEK2. **p < 0.01 and ***p < 0.001.

The diagnostic model showed good prediction ability. Among the five genes involved in the diagnosis formula, the coefficients of three genes (B3GNT3, GALNT7, and PLEK2) were positive, indicating that the higher the expression level of these genes, the higher the likelihood of being diagnosed with cancer. These three genes might play an oncogene role in lung cancer. To prove this, we respectively downregulated the expression of B3GNT3, GALNT7, PLEK2 in A549 and H1299 cells via RNAi treatment (Supplementary Figure S2). As expected, the silencing of these genes inhibited lung cancer cell proliferation and growth (Figures 3A–D, B3GNT3, Figures 3E–H, GALNT7, Figure 3I-L, PLEK2).

### Association Between MATH and Immune Infiltration Pattern

First, to explore the overall immune activity of the 389 early-stage LUAD patients in TCGA cohort, the enrichment abundance of 29 immune-related signatures was quantified using ssGSEA. As shown in the heatmap (Supplementary Figure S3A), the 389 patients were assigned to three different immune subtypes according to hierarchical clustering. The immune cluster 1, containing 134 (34.4%) patients, had the low enrichment level; the immune cluster 2, containing 74 (19%) patients, had the highest enrichment scores; and the immune cluster 3, containing 181 (46.5%) patients, was characterized by the medium enrichment level. Afterward, immune cell infiltration pattern was evaluated by the TIMER platform and ESTIMATE algorithm. The immune cluster 2 had the highest immune scores and stromal scores (Supplementary Figure S3E-F), indicating its high immunity, while immune cluster 1 showed the opposite. Six immune cell abundance (including B cell, macrophage, dendritic cell, neutrophil, CD4 T cell, and CD8 T cell, quantified by the TIMER platform) showed a gradual decrease from immune cluster 2 to immune cluster 3 to immune cluster 1 (Supplementary Figure S3B). Hence, immune clusters 1–3 were defined as low-immunity group, high-immunity group, and medium-immunity group, respectively. By using the OCLR algorithm built by Lian et al. (2019), stemness index (mRNAsi) of 389 LUAD patients was calculated based on gene expression data. However, mRNAsi was not different among the three groups (Supplementary Figure S3C, Kruskal–Wallis test, *p*-value = 0.61).

**FIGURE 4:**
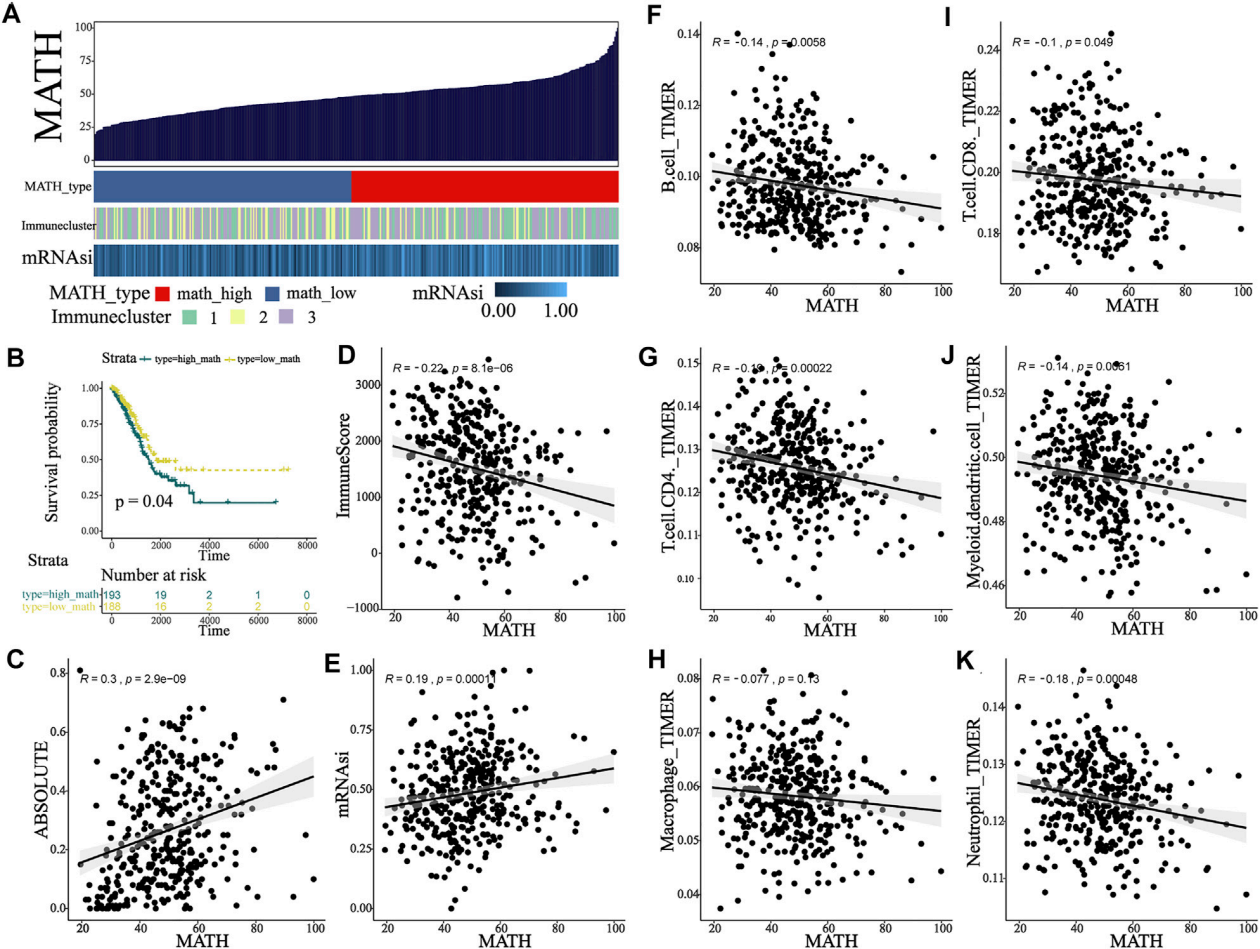
MATH associated with TIME and clinical outcome in early-stage LUAD patients. **(A)** Calculation of the MATH score in the main cohort and an overview of association between MATH and mRNAsi and immunity. **(B)** Survival analysis indicated significantly better overall survival in the low-MATH group. **(C)** Association between MATH and subclonal genome fraction quantified by ABSOLUTE. **(D)** Association between MATH and immune-score quantified by ESTIMATE. **(E)** Association between MATH and stemness index calculated by OCLR. **(F–K)** Association between MATH and tumor-infiltrating immune cells, including B cell, CD4 T cell, macrophage, CD8 T cell, dendritic cell, and neutrophil.

According to the previous study (Rocco, 2015), the heterogeneity in the tumor led to differences among mutated loci in terms of the fraction of sequence reads that show a mutant allele. The ratio of the width to the center of the distribution of mutant allele fractions, which is defined as MATH (mutant-allele tumor heterogeneity), is a reflection of the dispersion of variant allele frequencies, thus serving as a measure of intratumor heterogeneity. The MC3 file for TCGA-LUAD was analyzed, and MATH score for 389 patients was calculated using “inferHeterogenetiy” function (“maftool” package in R, Figure 4A). Then, we investigated the relationship between MATH score and ABSOLUTE score. A significant positive correlation between MATH and ABSOLUTE was observed (Figure 4C, *p* < 0.001), indicating that the MATH score could well reflect the ITH, so the MATH score was used to measure intratumor heterogeneity in our study. Notably, the MATH score seems to be negatively correlated with immune infiltration: immune cluster 2, which was defined as the high-immunity group, had the lowest MATH score as compared to other clusters (Supplementary Figure S3D, Kruskal–Wallis test, *p* = 0.005 and 0.074, respectively); and general negative correlation was observed between the MATH score and the abundance of immune cells (Figures 4D,F–K), which supported the notion that tumor-infiltrating immune cells shape the ITH. In addition, a positive correlation between mRNAsi and the MATH score was observed (Figure 4E, *p* < 0.001). To explore the potential link between the MATH score and clinical outcome, survival analysis was performed, and the optimal cutoff point of MATH score was determined by X-tile. Kaplan–Meier plot indicated that patients classified into the high-MATH group tended to have a worse overall survival outcome (Figure 4B, Log-rank test, *p* = 0.04). Briefly, the abovementioned results indicated that the MATH score was negatively correlated with tumor immune infiltration, and high ITH led to worse clinical outcome.

### Identification of Two MATH-Based Subtypes With Distinct Characteristics

**FIGURE 5:**
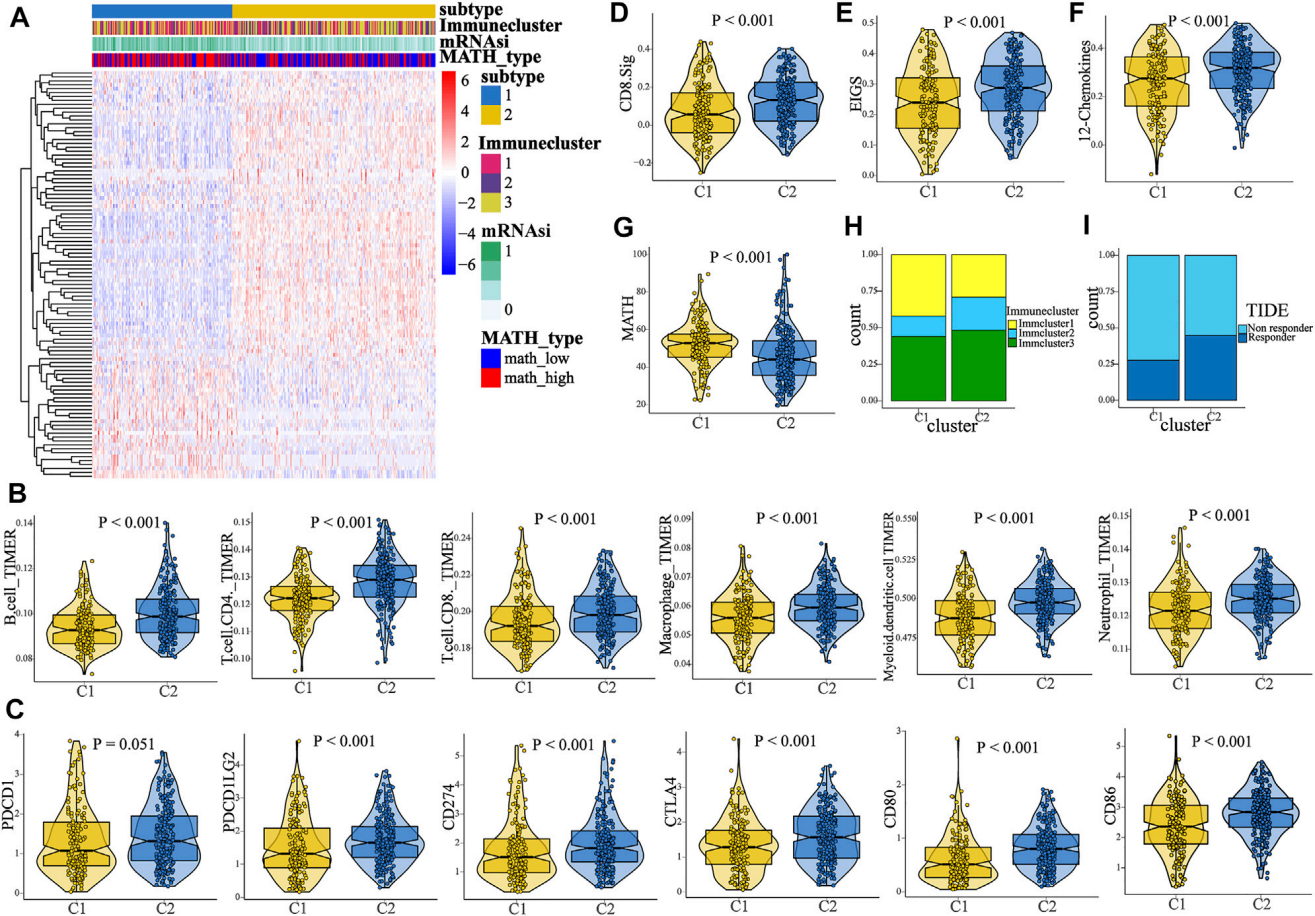
Identification of two MATH-based clusters with distinct characteristics and survival outcome. **(A)** Heat map showed the result of the unsupervised consensus clustering based on the 104 DEGs, and two clusters were identified. **(B)** Comparison of the abundance of B cell, CD4 T cell, CD8 T cell, macrophage, dendritic cell, and neutrophil between C1 and C2. **(C)** Comparison of the expression level of PD-1/PD-L1/PD-L2/CTLA-4/CD80/CD86 between C1 and C2. **(D–F)** Comparison of the enrichment level of three immunotherapy-related signatures between C1 and C2, including CD8. sig, EIGS (expanded immune gene signature), and 12 chemokines sig. **(G)** Patients in C1 had a higher MATH score than patients in C2. **(H)** Distinct proportions of different immune clusters in C1 and C2, indicating the different immunity between the two subtypes. **(I)**. TIDE algorithm predicted the likelihood of response to immunotherapy in C1 and C2.

Since survival difference was observed between the high- and low-MATH groups, we conducted differential gene expression analysis. A total of 104 DEGs were identified (Supplementary Figure S4A), which were defined as MATH-related DEGs. To unveil the MATH subtype, we further performed unsupervised consensus clustering (K-means) for early-stage LUAD patients based on the expression patterns of MATH-related DEGs (Supplementary Figure S4B, Supplementary Table S4). Thus, 389 early-stage LUAD patients were classified into cluster 1 (159 patients, 40.9%) and cluster 2 (230 patients, 59.1%) (Figure 5A), where cluster 1 tended to have a higher mRNAsi and MATH score (Figure 5G, Supplementary Figure S4C-D). Subsequently, immune cell abundance was compared between cluster 1 and cluster 2 (Figure 5B), and the six immune cells (B cell, CD4 T cell. CD8 T cell, macrophage, dendritic cell, and neutrophil, quantified by TIMER) were more abundant in cluster 2. The expression level of immune checkpoint molecules PD1 and CTLA-4, with its ligands (PDL1/PDL2, and CD80/CD86), was compared between C1 and C2. The results showed higher expression of these molecules in C2 (Figure 5C). In addition, immune cluster 2 had a higher proportion in C2 than C1, while immune cluster 1 had the opposite (Figure 5H).

**FIGURE 6:**
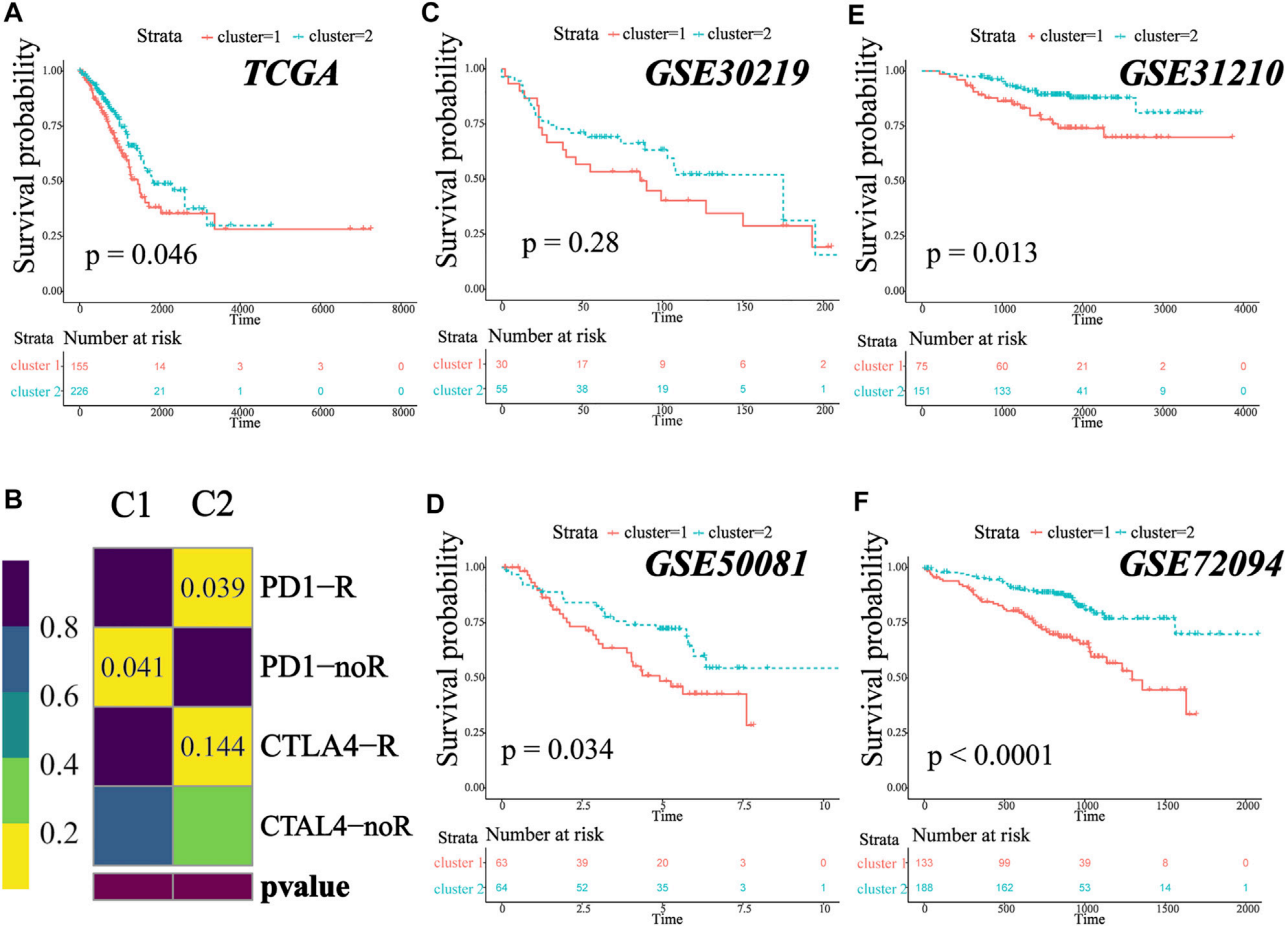
MATH-based subtype was a prevalent phenomenon in early-stage LUAD. **(A)**. Kaplan–Meier survival analysis revealed significantly better overall survival in patients in C2. **(B)** Submap analysis for predicting the likelihood of immunotherapy response for C1 and C2. **(C–F)** Different clinical overall survival outcomes between C1 and C2 in GSE30219, GSE50081, GSE31210, and GSE72094.

To predict the response to ICI (immune checkpoint inhibitor) therapy for patients in different clusters, the enrichment level of three immunotherapy-related signatures was quantified using ssGSEA. As compared to C1, patients in C2 had a significant higher enrichment of these signatures (CD8. sig, EIGS, 12-chemokines, Figures 5D–F), indicating that patients in C2 might have better response to ICI therapy. In addition, the TIDE algorithm was used to predict immunotherapy response. The result showed that the proportion of responders to immunotherapy in C2 was significantly higher than that in C1 (Figure 5I, chi-square test, *p* < 0.001). Next, subclass mapping was performed (“SubMap” module in GenePattern), and a melanoma immunotherapy cohort was set as the reference. It was found that patients in C2 might have better response to anti-PD1 or anti-CTLA4 therapy (Figure 6B). Afterward, we performed GSEA (gene set enrichment analysis) to identify hallmarks associated with different MATH clusters. GSEA results revealed that “Glycolysis”, “MYC_target”, “MTORC1 signaling”, and “PI3K-AKT-MTOR signaling” were enriched in C1, while “IL2-STAT5 signaling”, “inflammatory response”, “IFN-gamma response”, “P53 pathway”, and “TNFA signaling” were enriched in C2 (Supplementary Figure S4E-F). In brief, the abovementioned results indicated that samples in C2 had higher immune infiltration and immunogenicity, and patients in C2 had higher likelihood of response to immunotherapy than C1.

### The MATH-Based Clusters are Prevalent in Early-Stage Lung Adenocarcinoma

We analyzed the survival outcome of the two clusters, and the result revealed that patients in C2 had better overall survival (Figure 6A, TCGA cohort, log-rank test, *p* = 0.046). To investigate whether the two clusters were widespread in early-stage lung adenocarcinoma, we performed the same unsupervised consensus clustering (based on MATH-related DEGs) in GSE30219 (N = 85), GSE31210 (N = 226), GSE50081 (N = 127), and GSE72094 (N = 334). In all four independent validation sets, the early-stage LUAD patients could be classified into two groups, and patients in C1 tended to have a worse survival outcome (Figures 6C–F, log-rank test, *p*-value GSE30219: 0.28; GSE31210: 0.013; GSE50081: 0.034; GSE72094: <0.0001), which was consistent with our previous result. The survival difference did not reach statistical significance in the GSE30219 cohort, which might be due to the small sample size. To prove that the clusters generated in the validation cohorts were the same as those in TCGA cohort, we further performed submap analysis. As shown in the figure, the C1/C2 clusters of the validation cohorts could be well-mapped into the C1/C2 clusters of TCGA cohort (Supplementary Figure S5, all *p*-value and FDR <0.01). The result revealed that the MATH-based clusters are stable and widespread in early-stage lung adenocarcinoma, and patients in C2 tend to have a better survival outcome.

### Construction and Validation of the Cluster Predictor

**FIGURE 7:**
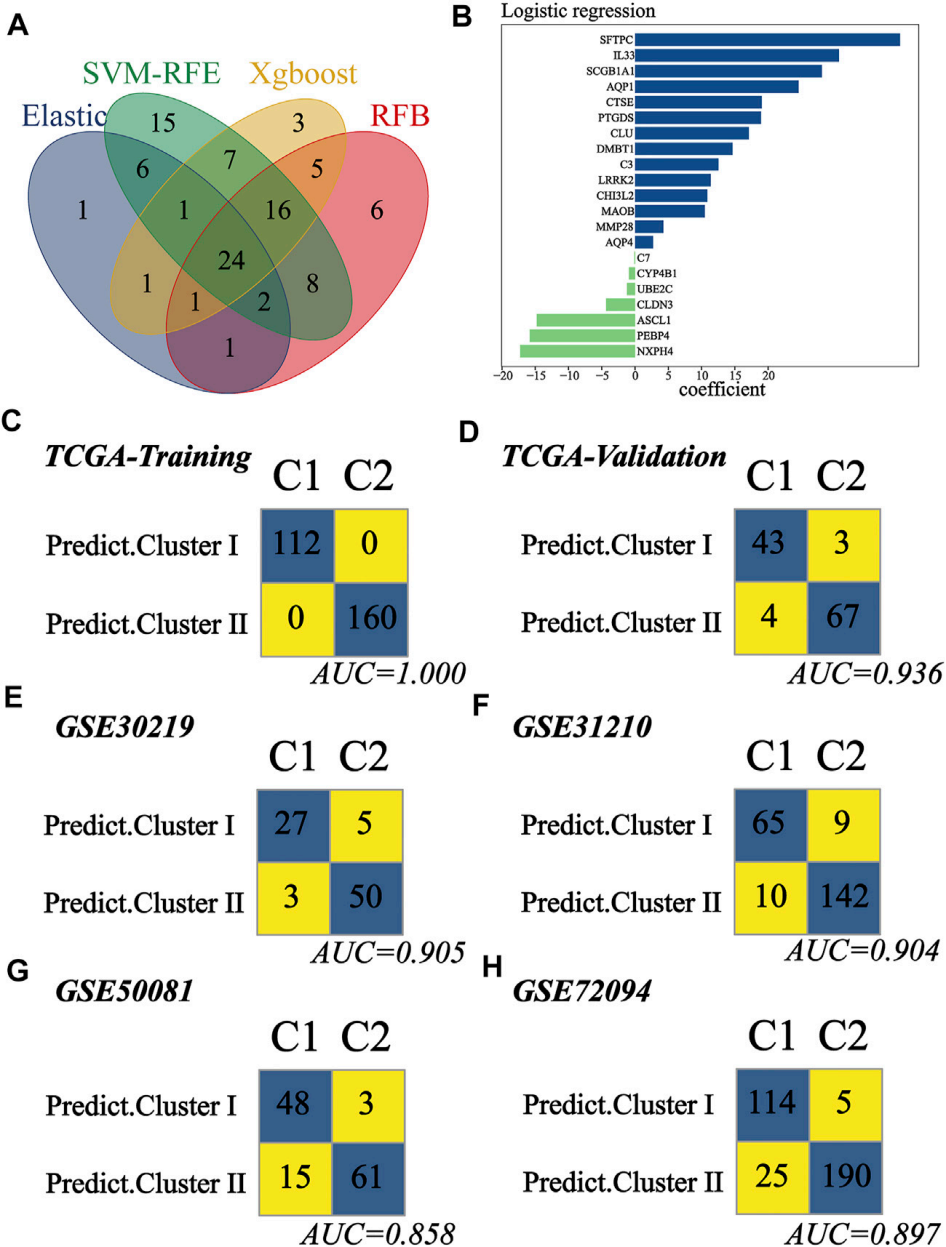
Construction and validation of the subtype predictor. **(A)** Venn diagram identified 24 genes shared by ElasticNet, SVM-RFE, RFB, and XGBoost machine learning. **(B)** Coefficient of the 21 common genes shared by all studying cohorts. The combination of these 21 genes was used to generate the subtype predictor using logistic regression. **(C–H)** Confusion matrices of binary results of the subtype predictor for TCGA training cohort, TCGA validation cohort, GSE30219, GSE31210, GSE50081, and GSE72094.

Since early-stage LUAD patients could be classified into two distinct clusters, we attempted to build a classifier to predict patient groups. TCGA cohort was divided into training and validation groups at a ratio of 7:3. GSE30219, GSE31210, GSE50081, and GSE72094 were used as external validation cohorts. In the training group, ML algorithms were applied to screen important features based on the expression file of 104 MATH-related DEGs, and a total of 37, 79, 58, and 63 genes were identified by ElasticNet, SVM-RFE, Xgboost, and RFB, respectively (Supplementary Figure S6, Supplementary Table S5). Twenty-four genes were found overlapping in different ML methods (Figure 7A). To construct the classifier, we reduced the 24 genes to 21 genes that were common to all datasets. The combination of the 21 genes was analyzed using logistic regression (Figure 7B), and we built the classifier. The confusion matrix for the training and validation groups and four external validation sets (GSE30219, GSE31210, GSE50081, and GSE72094) are shown in Figures 7C–H. In TCGA validation group, the accuracy, precision, recall, F1 score, and AUC for the classifier was 0.94, 0.93, 0.91, 0.92, and 0.94, respectively. In GSE30219, the accuracy, precision, recall, F1 score, and AUC for the classifier was 0.91, 0.84, 0.90, 0.87, and 0.91, respectively. In GSE31210, the accuracy, precision, recall, F1 score, and AUC for the classifier was 0.92, 0.88, 0.87, 0.87, and 0.90, respectively. In GSE50081, the accuracy, precision, recall, F1 score, and AUC for the classifier was 0.86, 0.94, 0.76, 0.84, and 0.86, respectively. In GSE72094, the accuracy, precision, recall, F1 score, and AUC for the classifier was 0.91, 0.96, 0.82, 0.88, and 0.90, respectively. The abovementioned result indicated that the classifier predictor we built had excellent performance and good generalization capability, and it could be instructive for the classification of early-stage LUAD patients.

### Epigenetic Differences Between C1 and C2

**FIGURE 8:**
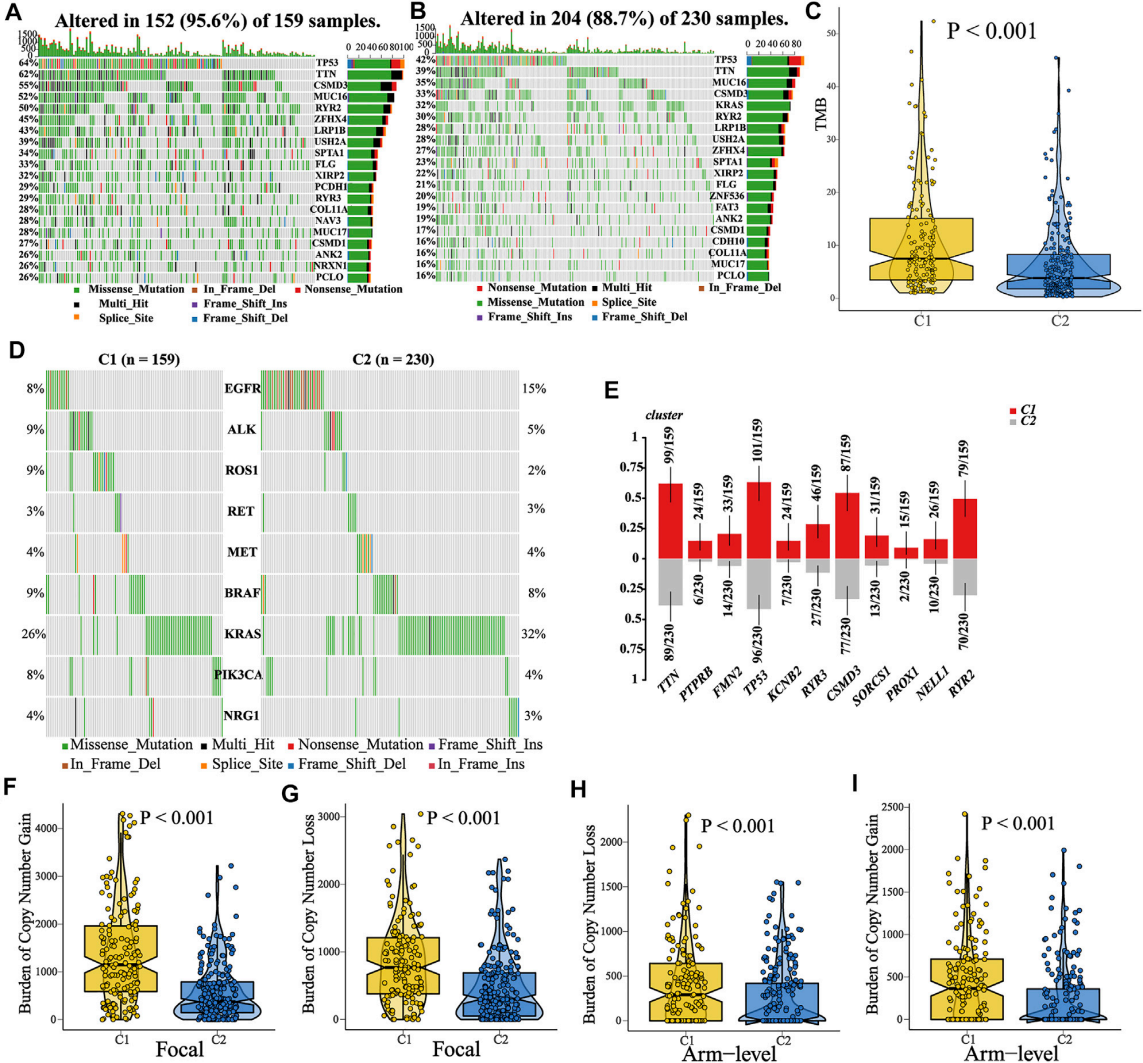
Comparisons of somatic variations and copy number alteration burden between C1 and C2. **(A–B)** Waterfall plots showed the top 20 mutated genes in C1 and C2. **(C)** Comparison of TMB between C1 and C2, and patients in C1 showed a higher TMB. **(D)** Mutation landscape of common driver genes of LUAD in C1 and C2. **(E)** Top 11 mutated genes enriched in patients in C1. **(F–I)** Comparison of CNA burden at the focal or arm level between C1 and C2.

Recent studies have shown that the epigenetic alterations are associated with immune evasion and tumor phenotype (Dawson and Kouzarides, 2012). Hence, we performed somatic mutation and CNA (copy number alteration) analysis to explore the difference between C1 and C2. Patients in C1 tended to have a higher TMB (tumor mutation burden) as compared to those in C2 (Figure 8C, Wilcoxon test, *p* < 0.001). The top 20 genes with the highest mutant frequency in each cluster are shown in Figures 8A–B, and there appeared to be little difference between C1 and C2. We further analyzed the mutation condition of known driver genes for LUAD and mutated genes enriched in C1. For the common driver genes in LUAD (including EGFR, ALK, ROS1, RET, MET, BRAF, KRAS, PIK3CA, and NRG1), only ROS1 had different proportion of mutation between C1 and C2 (Figure 8D, Supplementary Figure S7-8, Supplementary Table S6). In addition, there were 11 representative mutated genes enriched in C1 (namely, TTN, PTPRB, FMN2, TP53, KCNB2, RYR3, CSMD3, SORCS1, PROX1, NELL1, and RYR2, Figure 8E, Supplementary Figure S9, Supplementary Table S6). Afterward, we calculated and compared the CNA burden at the focal and arm level. Amplifications and deletions within chromosomal regions in each cluster were detected using GISTIC 2.0 (Supplementary Figure S10). Patients in the C2 had a lower burden of gain or loss at the arm or focal level (Figures 8F–I, Wilcoxon test; focal-level gain burden: <0.001; focal-level loss burden: <0.001; arm-level gain burden: <0.001; arm-level loss burden: <0.001), which was consistent with the previous notion that copy number alteration was related to immunotherapy resistance (Bassaganyas et al., 2020). All these underlying differences might be the cause of the different tumor phenotypes between C1 and C2. In addition, regarding the EGFR mutant, we found five types of mutations in these samples (Frame_shift_Del, In_Frame_Del, In_Frame_Ins, Missense_Mutation, and Nonsense_Mutation). Among these mutation types, most of them showed no difference between patients in C1 and C2 (Supplementary Table S7, Frame_shift_Del, C1: 0%; C2: 0.43%. In_Frame_Ins, C1: 0%; C2: 1.30%. Missense_Mutation, C1: 10.06%; C2: 12.17%. Nonsense_Mutation, C1: 0%; C2: 0.43%). Notably, at a 90% confidence interval, the frequency of EGFR in-frame deletion (In_Frame_Del) was higher in patients in C2 than that in C1 (C1:1.89%; C2: 6.09%). Previous studies have suggested that NSCLC patients with EGFR mutations are not suitable for immunotherapy. Meanwhile, patients with EGFR mutation who benefited from anti-PD (L)1 have been reported in some case reports. In our study, patients in cluster 2 tend to be more likely to benefit from immunotherapy, which may be due to the MATH-based classification. However, more studies are desired to screen the beneficiary population.

Then, we compared the expression of driver genes in different immune cells (B cells, CD4+T cells, CD8+T cells, DCs, and macrophages) between anti-PD1 responders and non-responders (treated with pembrolizumab or cemiplimab) in scRNA-seq data (GSE123813 cohort). The result showed that the responder B cells had a higher expression of BRAF and a lower expression of KRAS than the non-responders. Moreover, the expression of KRAS and PIK3CA in CD4+T cells was lower than that in the non-responders (Supplementary Table S8, Supplementary Figure S11).

### Identification of Potential Drugs for Patients in C1

**FIGURE 9:**
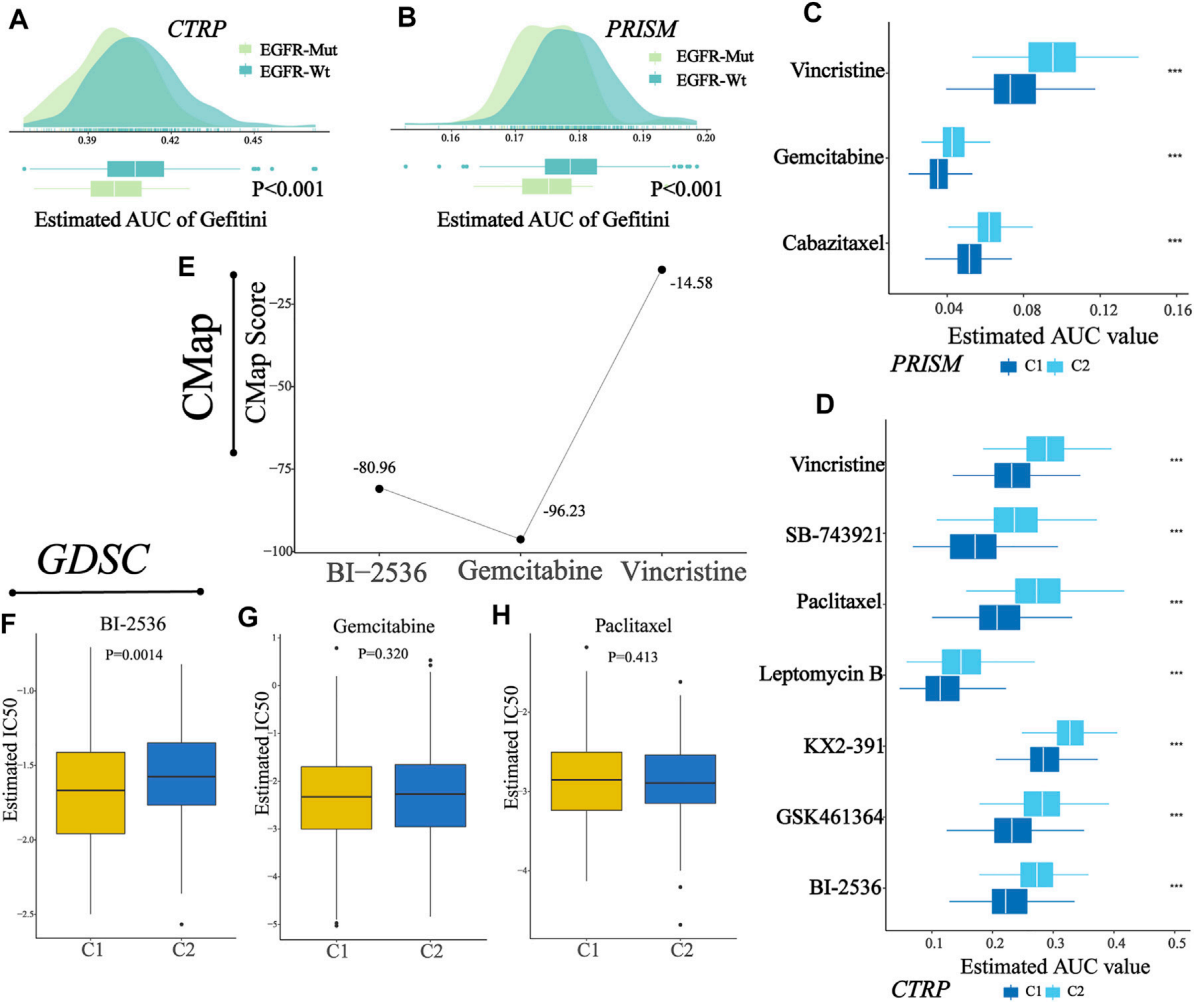
Identification of a potential agent with higher sensitivity for patients in C1. **(A–B)** Comparison of predicted Gefitini sensitivity between EGFR altered and unaltered groups. **(C)** Results of drug sensitivity analysis of three PRISM-derived compounds (vincristine, gemcitabine, and cabazitaxel). **(D)** Results of drug sensitivity analysis of seven CTRP-derived compounds (vincristine, SB-743921, paclitaxel, leptomycin B, KX2-391, GSK461364, and BI-2536). **(E)** CMap score of three candidate compounds (BI-2536, gemcitabine, and vincristine). **(F–H)** Comparison of estimated IC_50_ for BI-2536, gemcitabine, and paclitaxel in C1 and C2.

Two drug sensitivity profiles were generated after preprocessing the AUC files of CTRP and PRISM databases. Then, a ridge regression model was used (“pRRophetic” package in R) to predict the drug sensitivity of each sample in TCGA cohort (N = 389). For every sample, we obtained the AUC values of each compound contained in a certain database (CTRP or PRISM). Prior to further analysis, we attempted to demonstrate the reliability of drug sensitivity data. Patients in TCGA cohorts were assigned into two groups according to their EGFR alteration. We observed that patients with EGFR alteration showed lower AUC values of Gefitini than those without alteration both in CTRP and PRISM (Figures 9A–B), which was consistent with the clinical effect of Gefitini. Next, we identified the compounds (Figures 9C–D) with lower AUC values in C1 (log2FC > 0.2, *p*-value<0.05, Supplementary Table S9-10), including three PRISM-derived compounds (vincristine, gemcitabine, and cabazitaxel) and seven CTRP-derived compounds (vincristine, SB-743921, paclitaxel, leptomycin B, KX2-391, GSK461364, and BI-2536). These nine compounds all had lower AUC values in C1, indicating that patients in C1 had increased sensitivity to the corresponding treatment and might serve as the candidate potential therapeutic drug for patients in C1.

To explore the most reliable therapeutic compounds, we queried CMap (connectivity map) and GDSC (the Genomics of Drug Sensitivity in Cancer) and performed drug sensitivity analysis. As shown in the figure (Figure 9E), among candidate compounds identified before, BI-2536 and gemcitabine showed relatively low CMap scores (Fig, BI-2536: 80.96; gemcitabine: 96.23), indicating therapeutic potential; and among three compounds in GDSC overlapped in the candidate drugs, only BI-2536 showed a lower estimated IC_50_ in patients in C1 than that in C2 (Figure 9F-H, Wilcoxon test, *p*-value, BI-2536: 0.0014; gemcitabine: 0.3200; paclitaxel: 0.4130). The abovementioned results indicated that BI-2536 might be the promising potential treatment compound in patients in C1. The potential mechanism may be due to higher expression of PLK1 in the C1 cluster (Supplementary Table S11).

## Discussion

Intratumor heterogeneity (ITH) is a common phenomenon existing in all kinds of tumors (Andor et al., 2016). According to previous studies, increased ITH is correlated with poor clinical outcomes and has a negative association with immune infiltration (Hua et al., 2020). Besides, ITH has been reported to have an inverse association with T-cell immunoreactivity and sensitivity to immune checkpoint blockade (McGranahan et al., 2016). Thus, the analysis of ITH may create a new approach for cancer treatment. In our early-stage LUAD cohort, we used MATH as a measure of ITH quantification. We utilized TIMER and ESTIMATE algorithms to infer the immune infiltration pattern, and MATH was found to have negative correlation with the abundance of immune cells (including B cell, CD4 T cell, CD8 T cell, dendritic cell, macrophage, and neutrophils). Patients with high MATH tend to have a poor overall survival outcome. Also, by using the OCLR algorithm, we calculated the stemness indexes for each patient, and a positive correlation was found between MATH and cancer stemness. These results are consistent with those of previous studies (Miranda et al., 2019). In order to explore the ITH at a deeper level, an unsupervised consensus clustering was performed based on expression profiles of MATH-related DEGs. Early-stage LUAD patients in the main cohort had been classified into two groups (C1 and C2), and these two groups showed distinct characteristics. Specifically, patients in C2 had a lower MATH score than those in C1, indicating patients in C1 had higher ITH, while C1 had lower abundance of B cell, CD4 T cell, CD8 T cell, macrophage, dendritic cell, and neutrophil. To predict the likelihood of immunotherapy response in patients in C1 and C2, we compared the expression profiles of PD1/PDL1/PDL2 and CTLA-4/CD80/CD86 between C1 and C2, and three immunotherapy-related signatures were calculated using ssGSEA. The result revealed that patients in C2 had higher expression of immune checkpoint molecules and higher enrichment of immunotherapy-related signatures, indicating that patients in C2 were more likely to benefit from immunotherapy, while patients in C1 had the opposite. We further used the TIDE algorithm to predict the immunotherapy response, and it showed a consistent outcome. In addition, we observed the distinct clinical overall survival outcome between C1 and C2 across the main cohort and four independent validation cohorts (GSE30219, GSE31210, GSE50081, and GSE72094). Patients in C2 had an apparent survival advantage compared to patients in C1. In the end, drug analysis was conducted to find the potential treatment for high ITH patients (patients in C1). The results revealed that BI-2536 might have therapeutic potential for patients in C1.

In the current study, we have established two models using multiple machine learning algorithms (combined SVM-RFE, ElasticNet, XGBoost, and RFB): the early-stage LUAD diagnosis model and the early-stage LUAD classification predictor. For the former, the five-gene–based prognosis model (including B3GNT3, PLEK2, GALNT7, GRK5, and SLC39A8) reached an AUC of 0.982 in the internal validation cohort and AUC of 0.817 and 0.850 in the two external validation cohorts. B3GNT3 is an important member of B3GlcNacT family, and it was found important in the development of lung cancer (Sun et al., 2020). In addition, PLEK2 and GALNT7 have been reported to function as oncogenes in gall bladder cancer and colorectal cancer, respectively (Li et al., 2018; Shen H et al., 2019). The coefficients of these three molecules in our prognosis model are positive, which refers to cancerous tissue prediction, indicating that they might play important roles in the development of lung adenocarcinoma. Indeed, there are many studies on the prognostic model for early-stage LUAD. Generally, these models were based on the prognosis of LUAD patients. In our study, we explored the MATH-based LUAD subtypes and built a classification model. Compared with previous studies, (e.g., Lu et al. *A Prognostic Model for overall survival of patients with early-stage non–small cell lung cancer: a multicentre, retrospective study The Lancet digital health*; and Krzystanek et al. *A robust prognostic gene expression signature for early-stage lung adenocarcinoma. Biomarker Research*) (Krzystanek et al., 2016; Lu et al., 2020), there are some advantages and disadvantages of our research. For example, in the study by Lu et al., more omics data were included (eg. H&E–stained histology images, pathological parameters), which made the results more reliable. Moreover, these two research studies both used relatively large cohorts (Lu et alm multicenter, N = 1,057, Krzystanek et al., seven cohorts). In spite of these shortcomings, our study has some advantages. It is the first classification of early-stage LUAD based on MATH. We developed the classifier using current high-performance machine learning algorithms and verified its generalization ability. We tried to generate a small panel to help make a quick diagnosis and classification of LUAD at an early stage. However, its clinical translation value and application need further research. For the 21-gene–based classification model, we used several indices, such as accuracy, precision, recall, and F1 score, to measure the performance of our model across the internal validation cohort and four independent validation cohorts, and the result revealed its good predictive ability. It reached AUCs of 0.94, 0.91, 0.90, 0.86, and 0.90 in the internal validation cohort and independent validation cohorts (GSE30219, GSE31210, GSE50081, and GSE72094). Notably, the false-positive rate and the false-negative rate of our model were 4.29% and 8.51% in the internal validation cohort, 9.10% and 10.00% in GSE30219, 6.00% and 13.33% in GSE31210, 4.69% and 23.80% in GSE50081, and 2.56% and 18.00% in GSE72094, respectively indicating good generalization ability and application value. The main advantage of our model lies in its simplicity: a small gene panel could be designed for the detection and classification of early-stage LUAD. From this, we could get quick information about the feasibility of developing LUAD and which classification it belongs to. Since the two clusters of early-stage LUAD have distinct characteristics and prognosis, the model could help clinicians make appropriate treatment decisions. Directly, the combination of these molecules (shown in Figure 7B) could make a classification of NSCLC patients at an early stage. In our study, we elaborated on the clinical significance of this classification. In terms of individual molecules, most of them were studied in human cancers, including lung cancer (e.g., AQP1, AQP4, IL33, and PEBP4). For example, PEBP4 could promote the proliferation, migration, and EMT of lung cancer. Several studies suggested that aquaporin1 and aquaporin4 are related to the invasion of lung cancer. As for IL33, previous studies suggested that it could promote the occurrence and development of lung cancer. On the other hand, some studies suggested that it (IL33) could activate NK and CD8+T cells to suppress lung cancer. In general, these “candidate genes” could affect the biological function of lung cancer and further influence cancer phenotypes. In terms of clinical application, the combination of these genes is more meaningful. However, its clinical translation value and application need further research.

In conclusion, our study provided a new strategy for clinicians to make a quick preliminary assisting diagnosis of early-stage LUAD and make a patient classification at the intratumor heterogeneity level. Yet, this study has some unavoidable limitations and shortcomings. We found that patients in C1 exhibited a higher TMB and copy number burden and were enriched in certain mutations (such as TP53). However, we only depicted these characteristics in these involved clusters, and the correlation analysis did not reveal strong correlations. The causality was hard to be confirmed since our study is a retrospective analysis. The comparison of driver genes in immune cells between immunotherapy responders and non-responders was based on the sc-RNA seq dataset (GSE123813). Experimental validation needs to be further studied. In addition, though we used multiple cohorts to ensure the generalization ability of our findings, large sample clinical trials are needed to further confirm the clinical application value.

## Data Availability

The datasets presented in this study can be found in online repositories. The names of the repository/repositories and accession number(s) can be found in the article/Supplementary Material.
