# Supplementary material for: Integrated Analysis of MATH-Based Subtypes Reveals a Novel Screening Strategy for Early-Stage Lung Adenocarcinoma
Source: Front Cell Dev Biol. 2022 Feb 8;10:769711. doi: 10.3389/fcell.2022.769711 (PMC8861524; doi:10.3389/fcell.2022.769711)
Supplement: Supplementary file 2 [file Table1.DOCX]

**Table S1. Clinical information of early-stage LUAD patients in validation cohorts.**

| **Characteristics** |  | GSE30219 (N=85) | GSE31210 (N=226) | GSE50081 (N=127) | GSE72094 (N=334) |
| --- | --- | --- | --- | --- | --- |
| **Age** | Age>60 | 42 | 118 | 108 | 277 |
|  | Age<=60 | 43 | 108 | 19 | 57 |
| **Gender** | Female | 19 | 121 | 62 | 179 |
|  | Male | 66 | 105 | 65 | 155 |
| **Stage** | Stage i | 81 | 168 | 92 | 265 |
|  | Stage ii | 4 | 58 | 35 | 69 |
