# Supplementary material for: Integrated Analysis of MATH-Based Subtypes Reveals a Novel Screening Strategy for Early-Stage Lung Adenocarcinoma
Source: Front Cell Dev Biol. 2022 Feb 8;10:769711. doi: 10.3389/fcell.2022.769711 (PMC8861524; doi:10.3389/fcell.2022.769711)
Supplement: Supplementary file 3 [file DataSheet1.PDF]

Figure S1

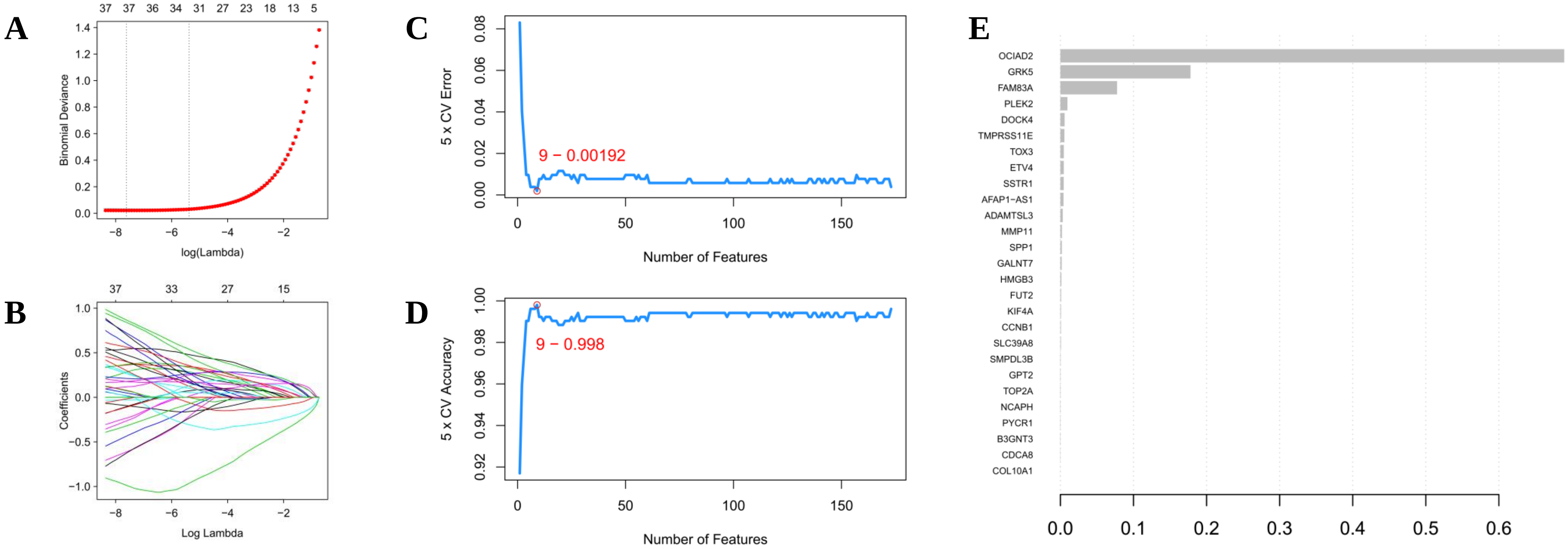

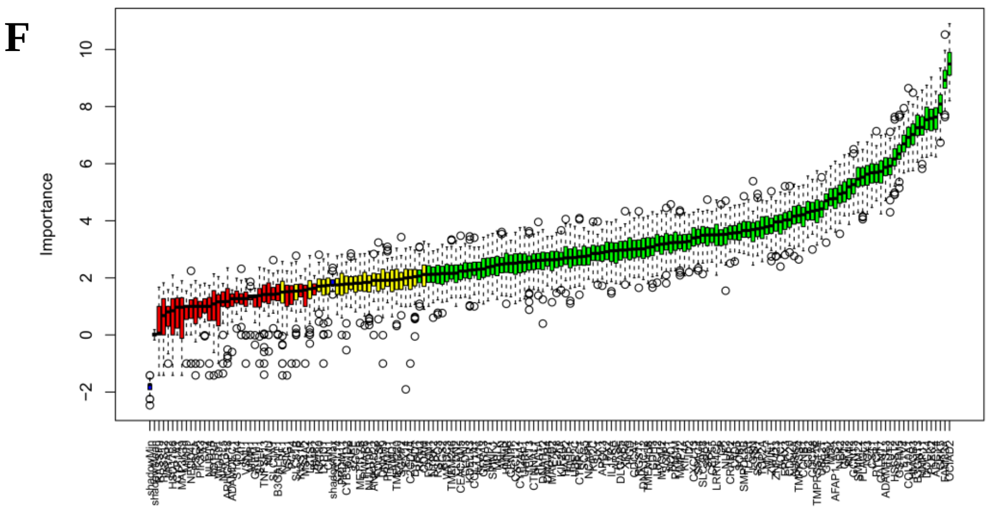

G

GSE30219 (N=99)	Non-cancerous tissue	Cancerous tissue	
Predict non-cancerous tissue	12	19	
Predict cancerous tissue	2	66	Totals
Totals	14	85	99
AUC			0.817

GSE31210 (N=246)	Non-cancerous tissue	Cancerous tissue	
Predict non-cancerous tissue	20	68	
Predict cancerous tissue	0	158	Totals
Totals	20	226	246
AUC			0.850

Figure S1. Construction and validation of the diagnosis model using four machine learning algorithms. (A)-(B) The binomial deviance and coefficients plot of candidate genes determined by ElasticNet method. (C)-(D) The error and accuracy plot of candidate genes determined by SVM-RFE. (E). The candidate genes determined by XGBoost. (F) The candidate genes determined by RFB. (G). The confusion matrices and AUCs of diagnosis model in GSE30219 and GSE31210 cohorts.

Figure S2

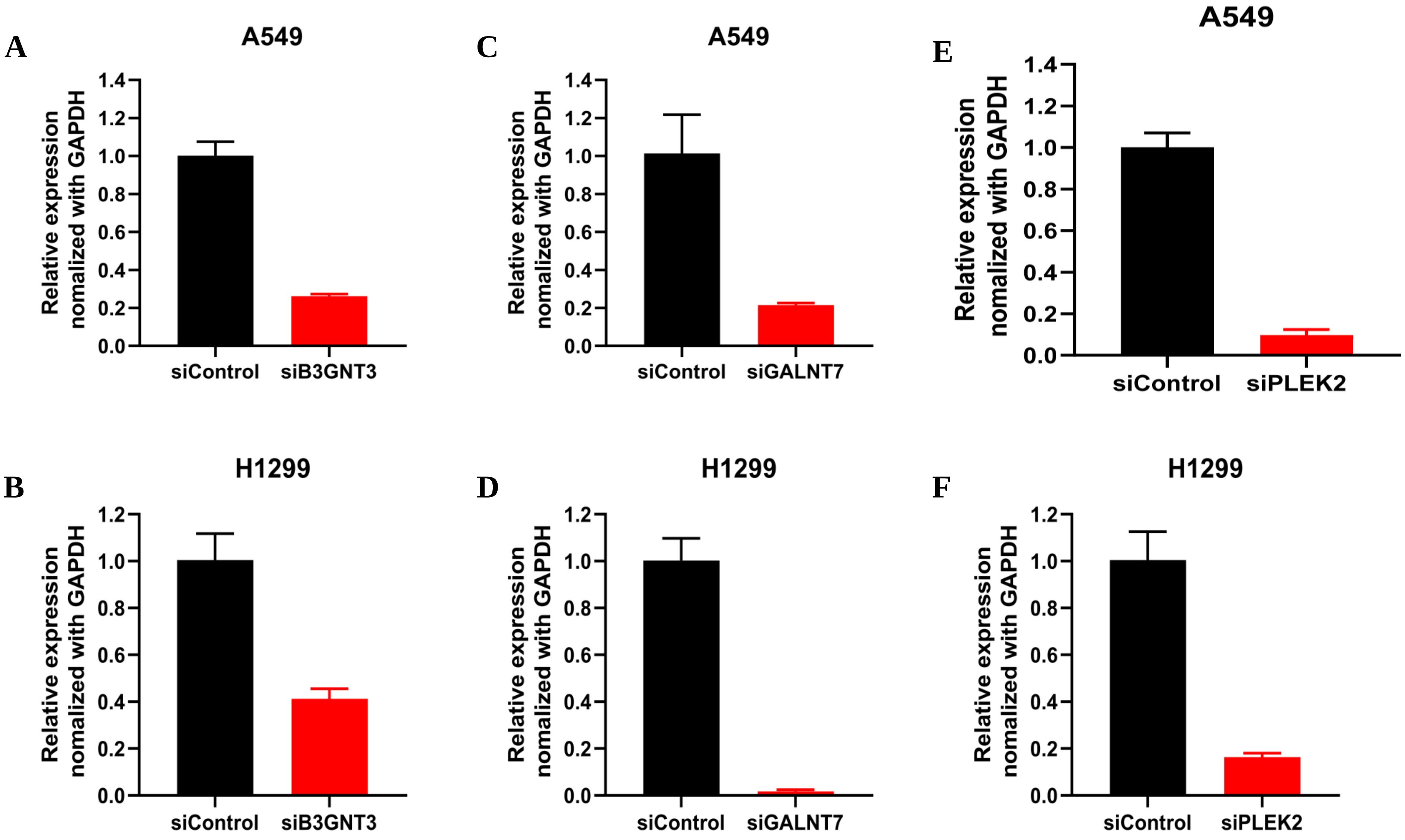

Figure S2 RT-qPCR revealed that target genes were efficiently knocked down in A549 and H1299 cells using siRNAs. (A)-(B). B3GNT3. (C)-(D). GALNT7. (E)-(F). PLEK2.

Figure S3

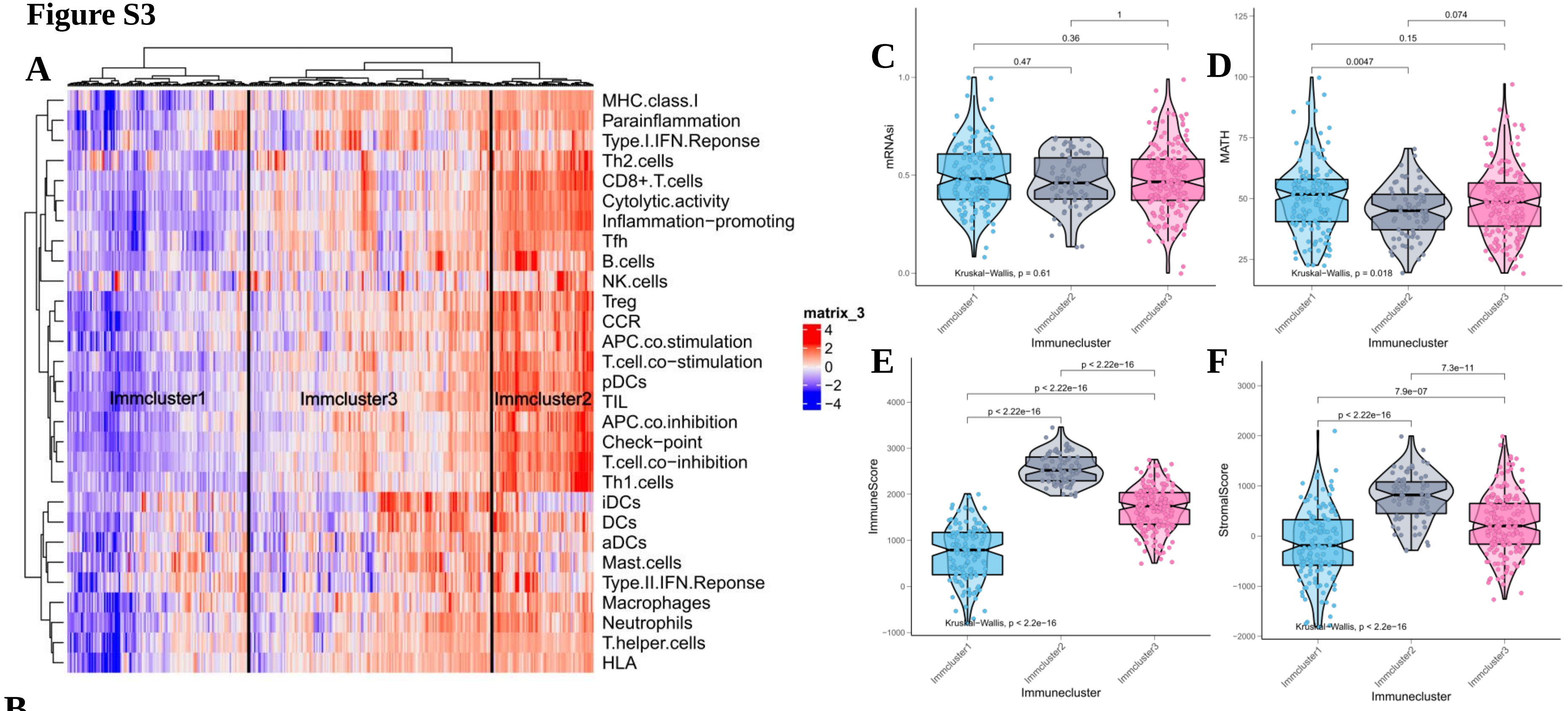

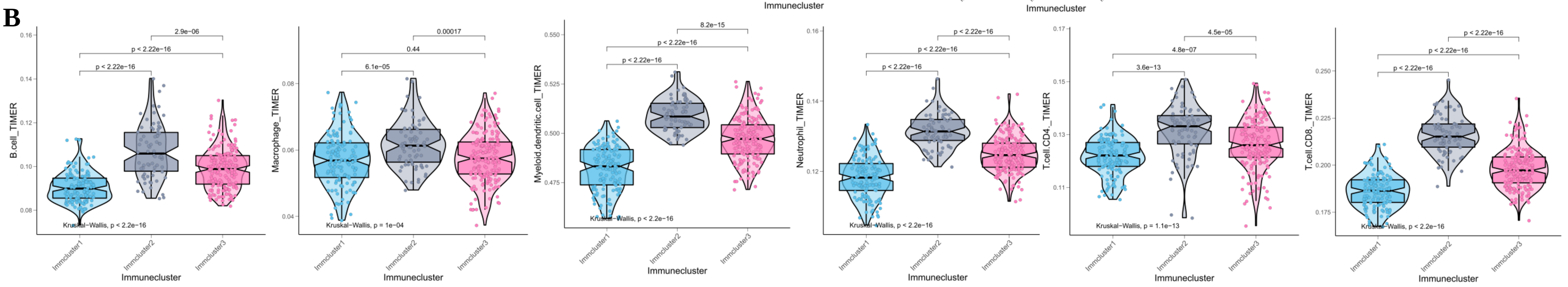

Figure S3. Identification of Immune-clusters. (A). Heat map showed the results of hierarchical clustering based on 29 ssGSEA of immune-related signatures. Patients has been classified into three different immunity groups. (B). Comparison of tumor infiltrating immune cells abundance among three immune-clusters. (C)-(F). Comparison of mRNAsi, MATH score, immune-score, and stromal score among three immune-clusters.

Figure S4

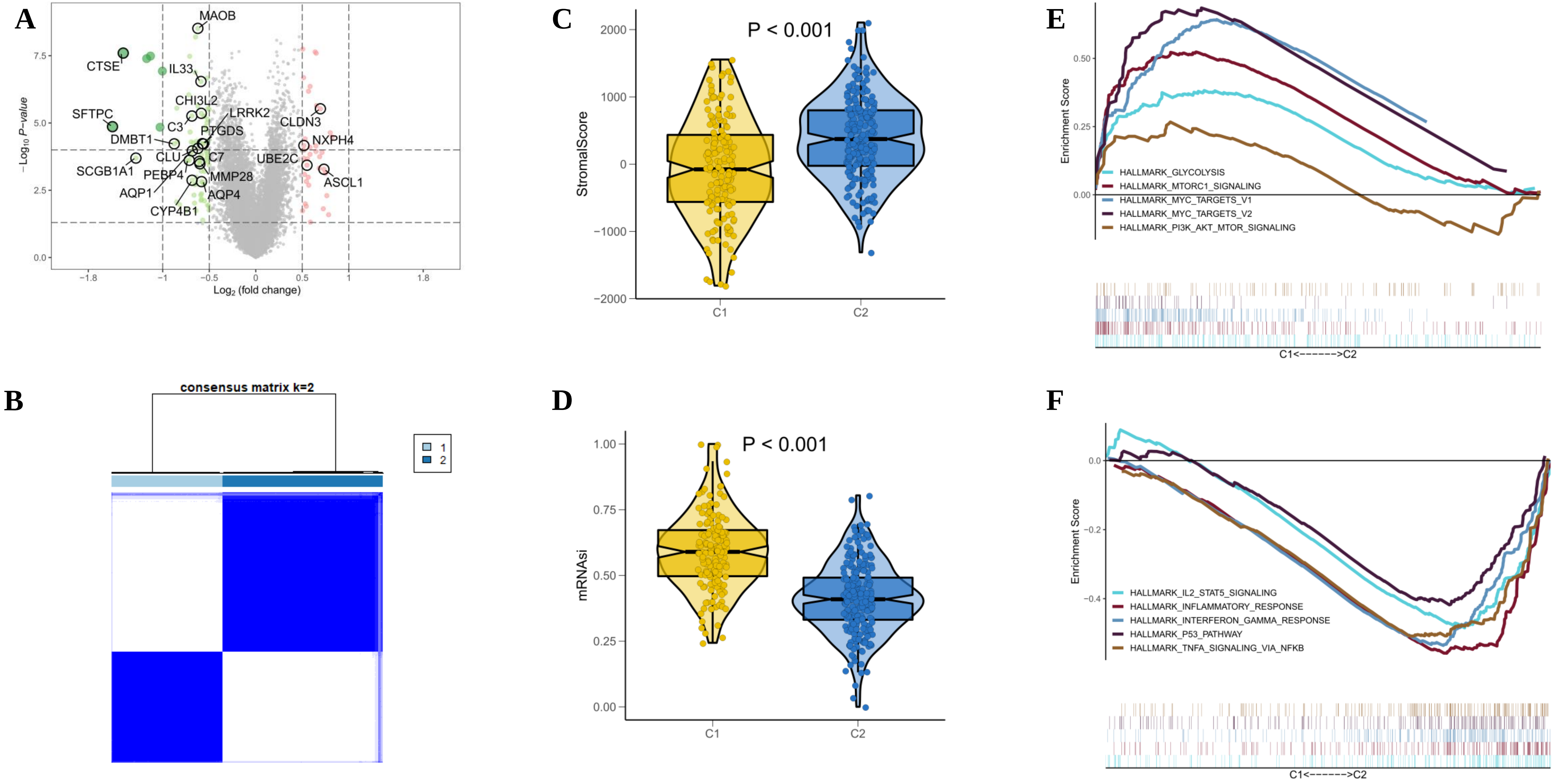

Figure S4. Identification of MATH-based clusters in early-stage LUAD. (A). The volcano plot showed differently expressed genes between high-MATH and low-MATH group. (B). Consensus clustering matrix when k=2 (Unsupervised consensus clustering based on 104 DEGs). (C)-(D). Comparison of stromal score and mRNAsi between C1 and C2. (E)-(F) GSEA results showed different hallmarks enriched in C1 or C2 groups.

Figure S5

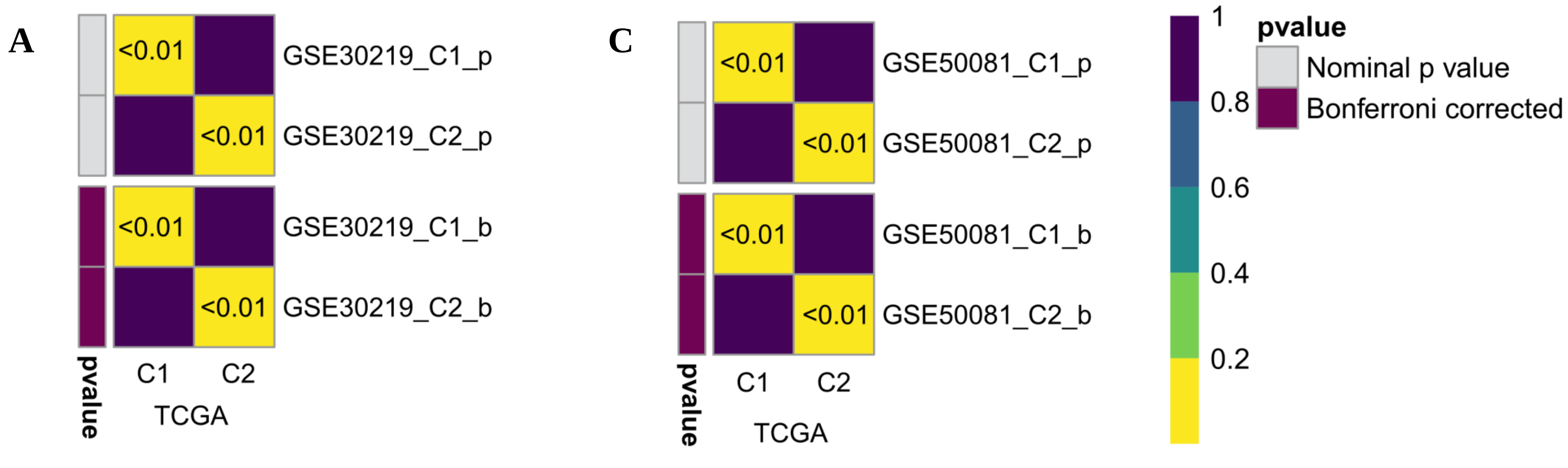

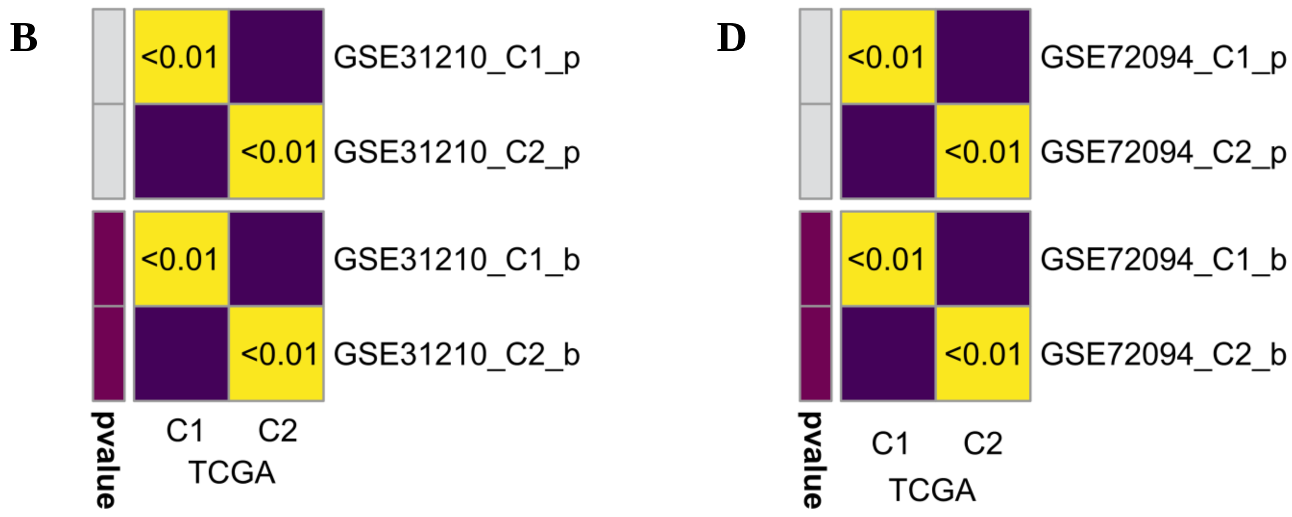

Figure S5. Subclass mapping results of two cluster in four independent validation cohorts. (A)-(D). The submap results of clusters in GSE30219, GSE31210, GSE50081, and GSE72094. The above results indicated that the clusters in the validation cohorts were the same as the TCGA cohort.

Figure S6

A

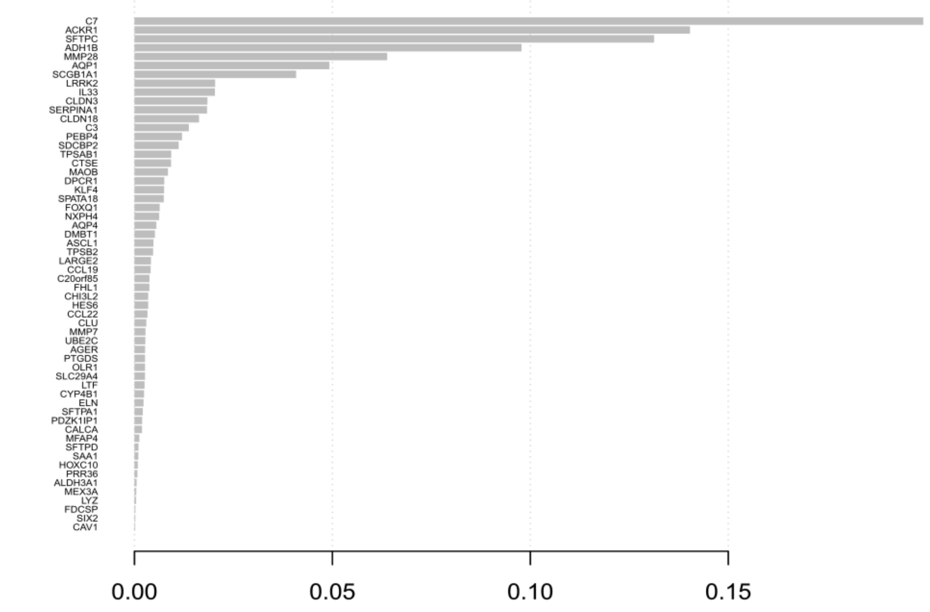

B

C

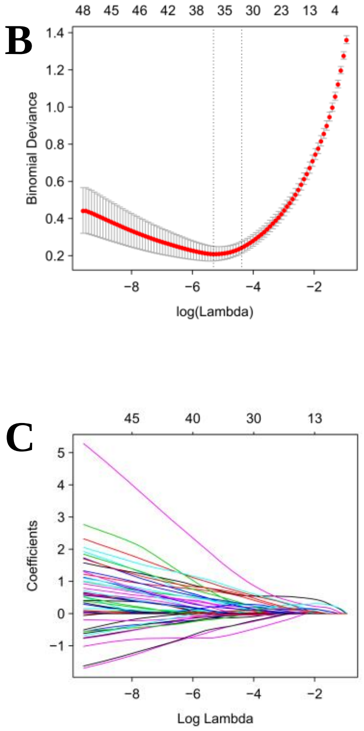

D

Genesymbol	ML	Genesymb	ML
SFTPC	SVM-RFE	SLC29A4	SVM-RFE
MAOB	SVM-RFE	CCL19	SVM-RFE
SCGB1A1	SVM-RFE	ITGB6	SVM-RFE
SFTPA1	SVM-RFE	MFAP4	SVM-RFE
SYT7	SVM-RFE	C3	SVM-RFE
IL33	SVM-RFE	ALDH3A1	SVM-RFE
TUBB2B	SVM-RFE	MMP28	SVM-RFE
CCL18	SVM-RFE	CAV1	SVM-RFE
AQP4	SVM-RFE	TNNC2	SVM-RFE
ACKR1	SVM-RFE	COL17A1	SVM-RFE
DMBT1	SVM-RFE	OLR1	SVM-RFE
PTGDS	SVM-RFE	SST	SVM-RFE
FGL1	SVM-RFE	CXCL1	SVM-RFE
AQP1	SVM-RFE	FHL1	SVM-RFE
FOS	SVM-RFE	MRC1	SVM-RFE
C20orf85	SVM-RFE	CSAG1	SVM-RFE
CLU	SVM-RFE	PEG10	SVM-RFE
CTSE	SVM-RFE	MEX3A	SVM-RFE
AGER	SVM-RFE	CLDN18	SVM-RFE
FCN3	SVM-RFE	MS4A1	SVM-RFE
ADH1B	SVM-RFE	PRR36	SVM-RFE
MAGEA6	SVM-RFE	SERPINA	SVM-RFE
LTB	SVM-RFE	CPA3	SVM-RFE
PEBP4	SVM-RFE	NXPH4	SVM-RFE
SDCBP2	SVM-RFE	SOX2	SVM-RFE
SBK1	SVM-RFE	SIX2	SVM-RFE
SFTPD	SVM-RFE	CST1	SVM-RFE
MUC5AC	SVM-RFE	TPSAB1	SVM-RFE
ASCL1	SVM-RFE	JCHAIN	SVM-RFE
ELN	SVM-RFE	LYZ	SVM-RFE
C7	SVM-RFE	PSCA	SVM-RFE
AADAC	SVM-RFE	TSPAN7	SVM-RFE
HES6	SVM-RFE	KRT6A	SVM-RFE
FOXA1	SVM-RFE	GFRA3	SVM-RFE
UBE2C	SVM-RFE	FOXO6	SVM-RFE
CLDN3	SVM-RFE	FDCSP	SVM-RFE
FOXQ1	SVM-RFE	CHI3L2	SVM-RFE
CYP4B1	SVM-RFE	CALCA	SVM-RFE
TPSB2	SVM-RFE	LRRK2	SVM-RFE
		SLC6A14	SVM-RFE

E

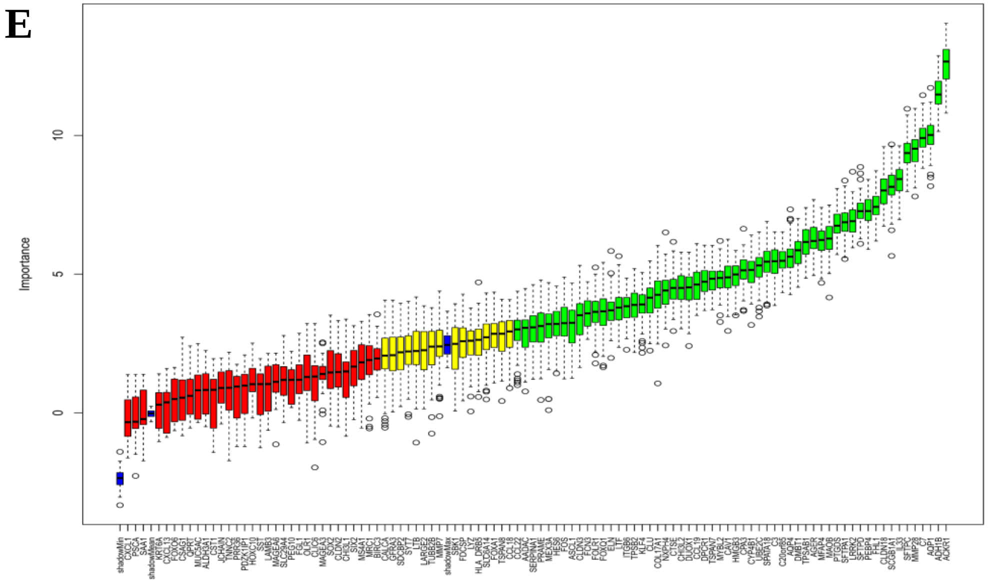

Figure S6. Construction and validation of the subtypes predictor using four machine learning algorithms. (A). The candidate genes determined by XGBoost. (B)-(C). The binomial deviance and coefficients plot of candidate genes determined by ElasticNet method. (D). The candidate genes determined by SVM-RFE. (E). The candidate genes determined by RFB.

Figure S7

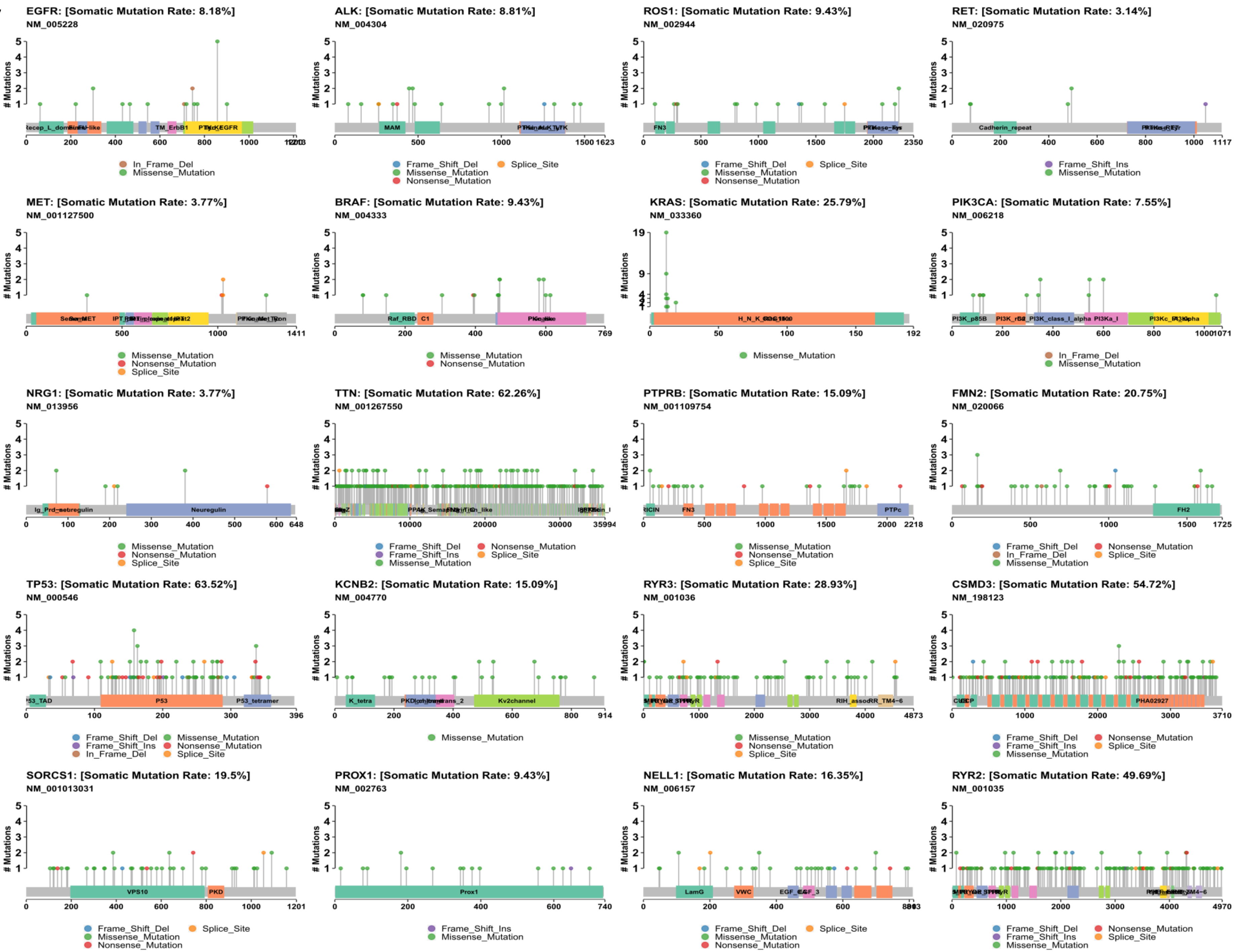

Figure S7. The mutation sites and types of several important genes in C1 patients.

Figure S8

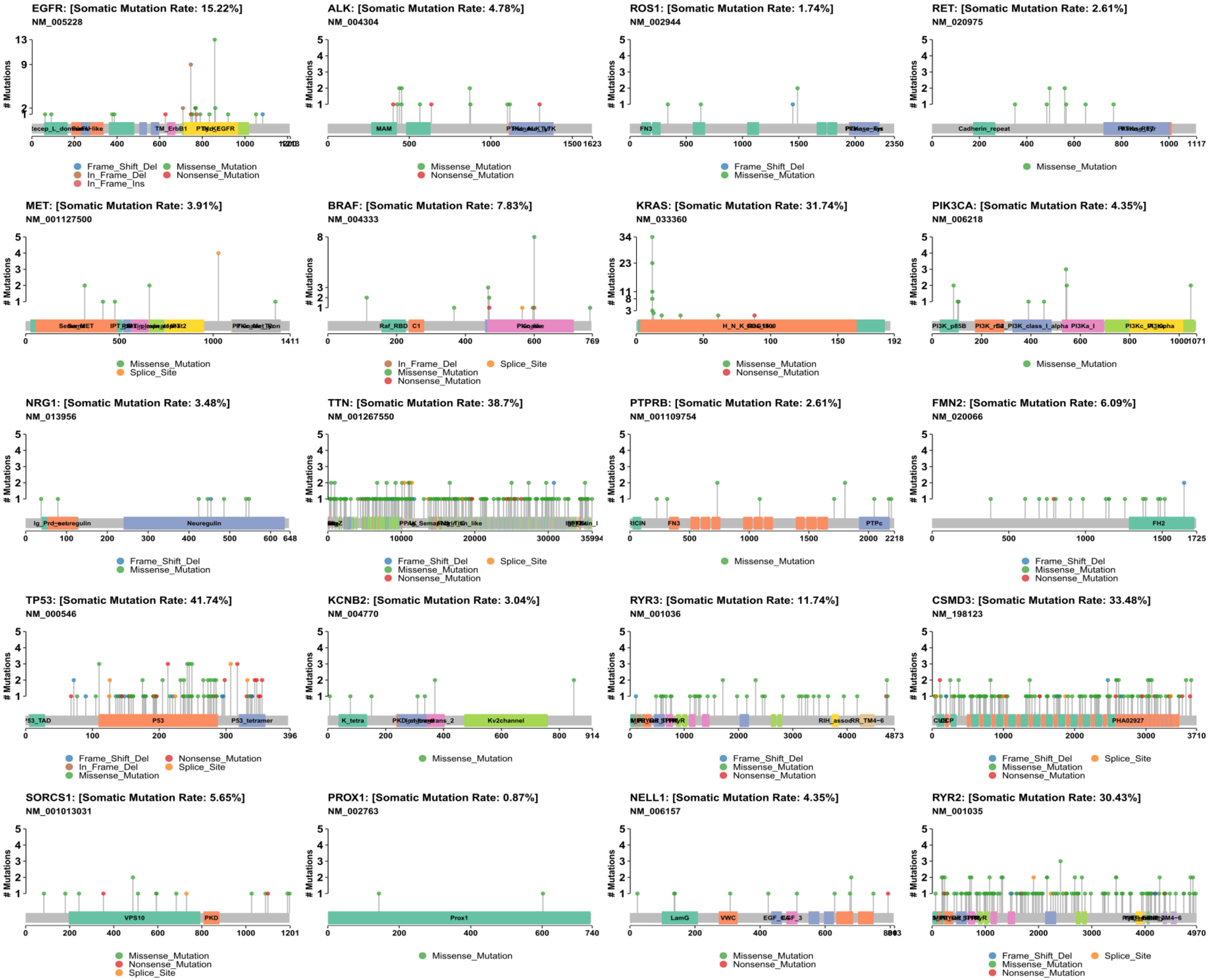

Figure S8. The mutation sites and types of several important genes in C2 patients.

Figure S9

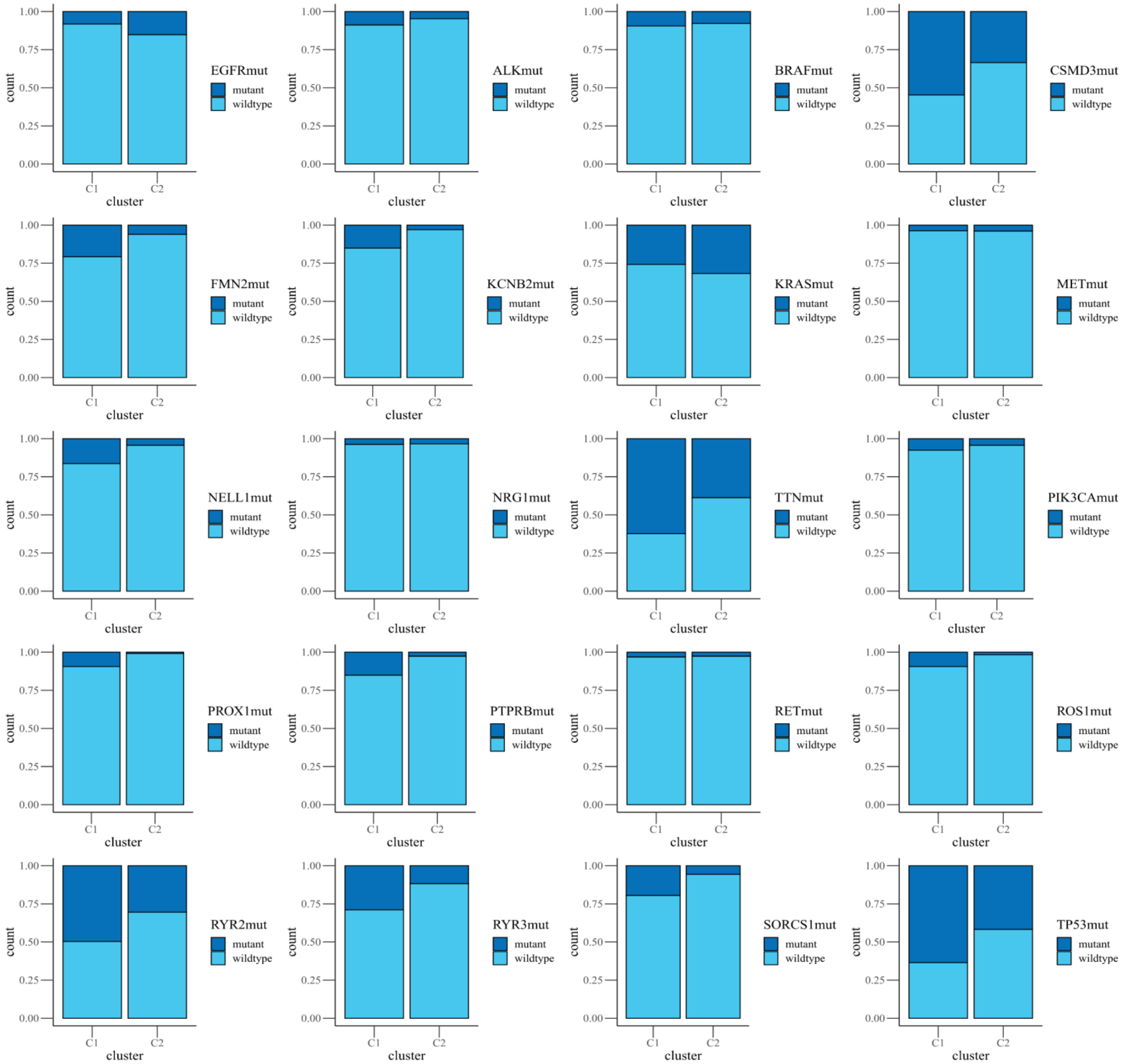

Figure S9. Comparison of the mutataion proportions of several important genes between C1 and C2.

Figure S10

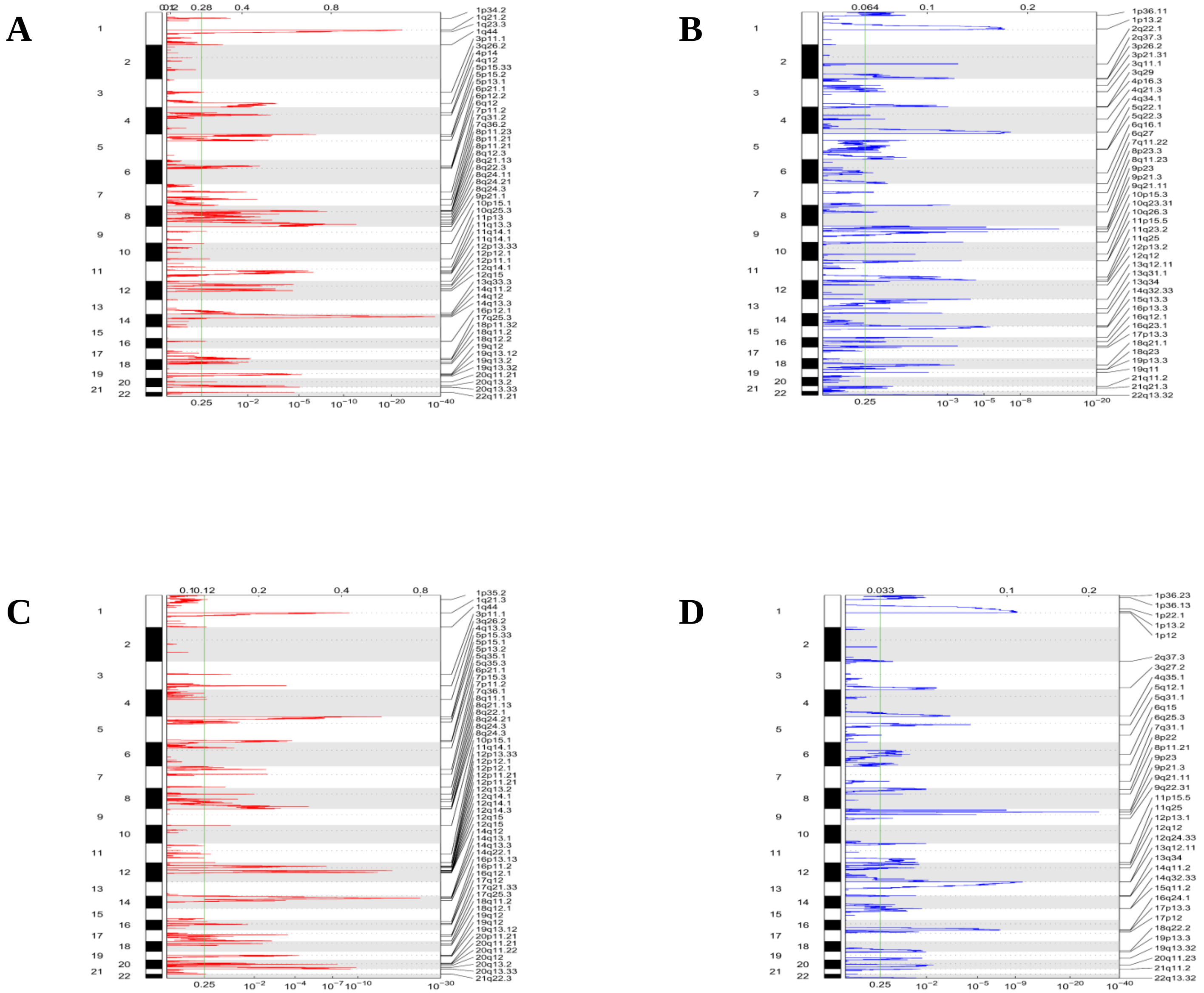

Figure S10. The copy number alterations across C1 and C2. (A)-(B) The detailed gene copy number amplification and deletion in the C1 group generated by GISTIC 2.0. (C)-(D). The detailed gene copy number amplification and deletion in the C2 group generated by GISTIC 2.0.

Figure S11

GSE123814 Single cell RNA sequencing Data

**Annotation**	**Maker(s)**
Myofibroblasts	ACTA2, MCAM, MYLK
T cells	CD3D, CD3G, CD3E
CD8 + T cells	CD8A, GZMA
Tregs	CD4, FOXP3
NK cells	KLRC1, KLRC3
B cells	CD19, CD79A
Plasma cells	SLAMF7, IGKC
Macrophage	FCGR2A, CSF1R
Dendritic	FLT3
Fibroblasts	COL1A2
CAFs cancer-associated fibroblasts	FAP, PDPN
Malignant cells	EPCAM
Endothelial cells	PECAM1, VWF

Comparison between Responders to anti-PD1 therapy and Non-Responders to anti-PD1 therapy

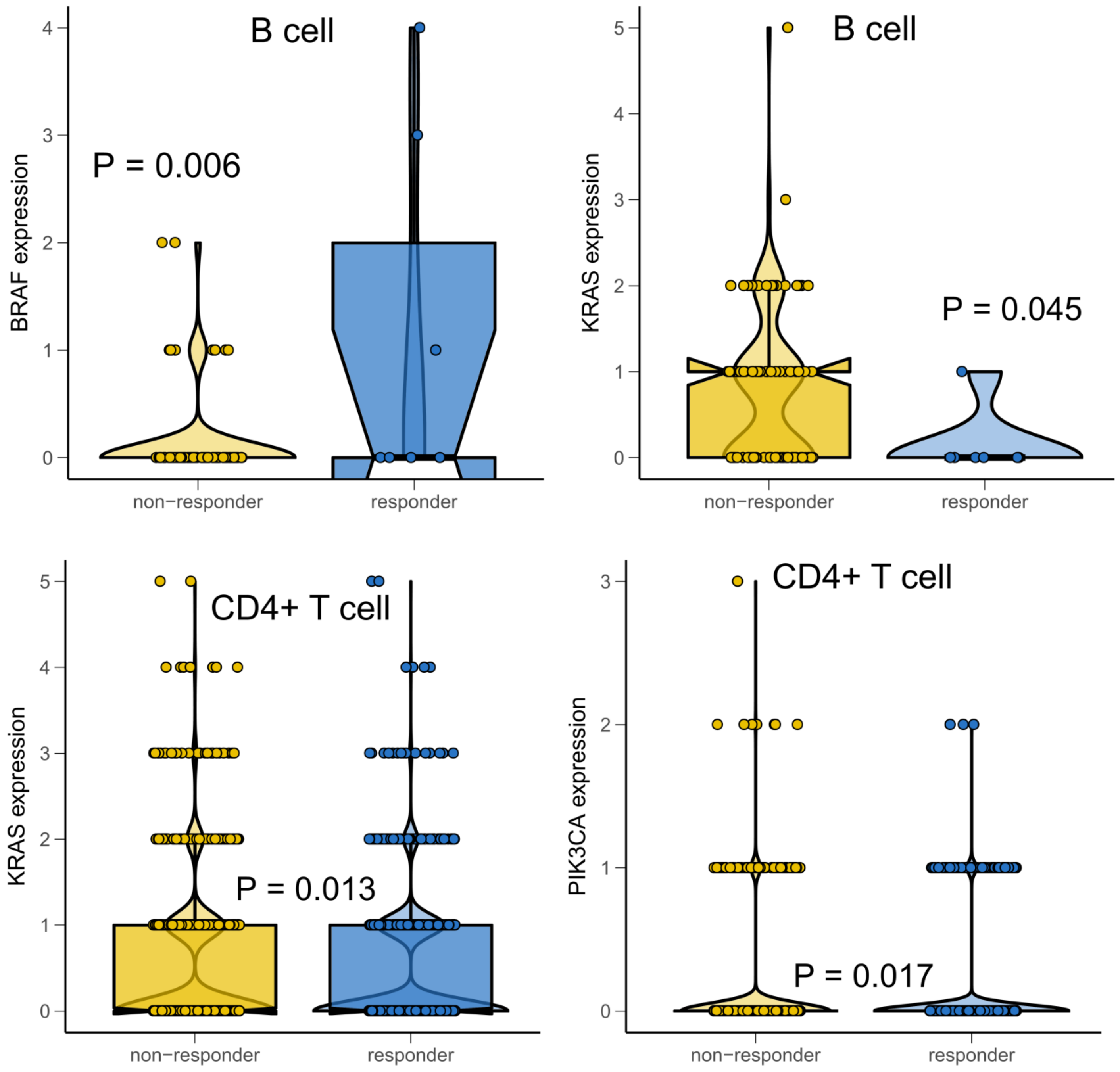

Figure S11. The expression of drive genes across different immune cells in immunotherapy cohort.
